# Defining developmental trajectories of prosensory cells in human inner ear organoids at single-cell resolution

**DOI:** 10.1242/dev.201071

**Published:** 2023-06-29

**Authors:** Yoshitomo Ueda, Takashi Nakamura, Jing Nie, Alexander J. Solivais, John R. Hoffman, Becca J. Daye, Eri Hashino

**Affiliations:** ^1^ Department of Otolaryngology-Head and Neck Surgery, Indiana University School of Medicine, Indianapolis, IN 46202, USA; ^2^ Department of Otolaryngology-Head and Neck Surgery, Kyoto Prefectural University of Medicine, Kyoto 602-8566, Japan; ^3^ Stark Neurosciences Research Institute, Indiana University School of Medicine, Indianapolis, IN 46202, USA; ^4^ Department of Anatomy and Cell Biology, Indiana University School of Medicine, Indianapolis, IN 46202, USA; ^5^ Department of Pharmacology and Toxicology, Indiana University School of Medicine, Indianapolis, IN 46202, USA

**Keywords:** Inner ear, Human, Prosensory cells, Hair cells, scRNA-seq, Developmental trajectory

## Abstract

The inner ear sensory epithelia contain mechanosensitive hair cells and supporting cells. Both cell types arise from SOX2-expressing prosensory cells, but the mechanisms underlying the diversification of these cell lineages remain unclear. To determine the transcriptional trajectory of prosensory cells, we established a *SOX2*-2A-ntdTomato human embryonic stem cell line using CRISPR/Cas9, and performed single-cell RNA-sequencing analyses with *SOX2*-positive cells isolated from inner ear organoids at various time points between differentiation days 20 and 60. Our pseudotime analysis suggests that vestibular type II hair cells arise primarily from supporting cells, rather than bi-fated prosensory cells in organoids. Moreover, ion channel- and ion-transporter-related gene sets were enriched in supporting cells versus prosensory cells, whereas Wnt signaling-related gene sets were enriched in hair cells versus supporting cells. These findings provide valuable insights into how prosensory cells give rise to hair cells and supporting cells during human inner ear development, and may provide a clue to promote hair cell regeneration from resident supporting cells in individuals with hearing loss or balance disorders.

## Teaser

**Highlighted Article:** Longitudinal scRNA-seq analysis of human inner ear organoids identifies supporting cells as possible precursors of hair cells and upregulation of previously unrecognized early genes during hair cell differentiation.

## INTRODUCTION

During inner ear development, prosensory cells arise from a subpopulation of PAX2/PAX8-positive multipotent otic progenitors and give rise to mechanosensitive hair cells and supporting cells. The earliest prosensory cells are defined by the expression of SOX2, JAG1 and EPCAM, followed by the expression of P27^KIP1^ (*CDKN1B*), NGFR (*CD271*), HEY2 and PROX1, and the downregulation of JAG1 in the mouse cochlea (Dabdoub et al., 2008; Zak and Daudet, 2021; Roccio et al., 2018; Basch et al., 2011; Pan et al., 2013; Ohyama et al., 2010). Previous studies have shown that SOX2 is required for the proper development of sensory epithelia in the mouse inner ear (Pan et al., 2013; Kiernan et al., 2005). SOX2 is constitutively expressed, as a pluripotency marker, in undifferentiated human embryonic stem cells (hESCs), but its expression promptly declines upon germ layer differentiation (Ealy et al., 2016). Over the course of mouse inner ear development, SOX2 is expressed initially in both sensory and nonsensory regions in the otic vesicle, and later becomes confined to prosensory cells (Dabdoub et al., 2008; Kempfle et al., 2016; Steevens et al., 2019). After hair cell differentiation, SOX2 expression quickly subsides in type I hair cells in the vestibular end organ, and in inner and outer hair cells in the cochlea, but SOX2 remains highly expressed in type II hair cells in the vestibule and supporting cells in both vestibular and cochlear epithelia (Dabdoub et al., 2008; Kempfle et al., 2016; Stone et al., 2021; Lu et al., 2019).

As hair cells do not regenerate in the mammalian inner ear at any clinically relevant levels, intense research has been under way to identify genes essential for hair cell differentiation. *ATOH1* is one of the first genes revealed to be required for proper hair cell differentiation in the mouse cochleae (Woods et al., 2004; Bermingham et al., 1999; Liu et al., 2014a) and has been studied extensively. SOX2 directly binds the SOX transcription factor-binding site in the *ATOH1* regulatory region (Neves et al., 2012) and promotes its expression during hair cell differentiation (Ahmed et al., 2012; Kiernan et al., 2005). POU4F3 acts reciprocally with ATOH1 to post-translationally promote hair cell differentiation (Yu et al., 2021). Additionally, GFI1 indirectly enhances the ATOH1 transcriptional activity as part of a DNA-binding complex and directly represses the neuronal genes to promote hair cell differentiation and survival (Jen et al., 2022; Matern et al., 2020). Forced expression of *ATOH1*, alone or in combination with *GFI1* and/or *POU4F3*, was shown to promote hair cell differentiation through the transdifferentiation from supporting cells in the mouse cochlea *in vitro* (Lee et al., 2020a; Chen et al., 2021; Liu et al., 2012, 2014b; Kuo et al., 2015; Zheng and Gao, 2000). Despite the wealth of data on well-established candidate pioneer genes, exogenous expression of these candidate genes has mixed results (Chen et al., 2021; Walters et al., 2017; Yamashita et al., 2018; Lee et al., 2020b; Iyer et al., 2022), suggesting the existence of yet-to-be-identified genes that can drive the initiation of hair cell differentiation.

The goal of the present study is to identify previously unrecognized genes or gene sets that are upregulated at a very early stage of human hair cell differentiation. Recently developed protocols efficiently generate inner ear organoids from aggregates of human pluripotent stem cells (Koehler et al., 2017; Nie and Hashino, 2020; Ueda et al., 2022; Nie et al., 2022; Valk et al., 2022 preprint). The resultant organoids constitute layered vesicular epithelia harboring functional hair cells on the luminal surface of the epithelia. Our protocols are intrinsically skewed for deriving vestibular epithelia without any cochlear cell types. Moreover, the majority of hair cells in the organoids appear to be type II vestibular hair cells (Koehler et al., 2013, 2017). By taking advantage of sustained SOX2 expression in otic progenitors, prosensory cells, supporting cells and type II vestibular hair cells in this system, we have performed a longitudinal study of transcriptomic trajectories of *SOX2*-expressing cells in human inner ear organoids, with particular focus on a branching point towards hair cell versus supporting cell specification.

## RESULTS

### Generation and validation of a *SOX2*^ntdTomato^ hESC reporter line

**Fig. 1.**
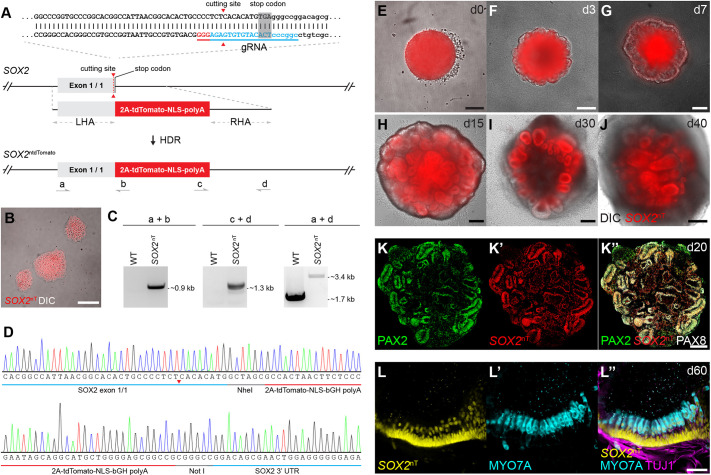
**Generation and validation of a *SOX2*^ntdTomato^ reporter hESC line.** (A) CRISPR knock-in design of *SOX2*-2A-tdTomato-NLS-polyA (*SOX2*^ntdTomato^ or *SOX2*^nT^). (B) Normal hESC morphology and *SOX2*^ntdTomato^ expression. (C) PCR genotyping of wild-type and *SOX2*^ntdTomato^ cell lines using primers shown in A suggests successful biallelic 2A-tdTomato-NLS-polyA knock-in. (D) Sanger sequencing chromatograms of the left and right junction areas between the insertion cassette and the genomic DNA. The red triangle indicates the Cas9 cutting site. (E-J) *SOX2*^ntdTomato^ fluorescence signals in inner ear organoids from d0-35. (K-K″) Immunofluorescence of *SOX2*^ntdTomato^ and otic progenitor markers PAX2 and PAX8 in d20 inner ear organoids. (L-L″) MYO7A^+^ *SOX2*^ntdTomato+^ hair cells innervated by TUJ1^+^ neuronal fibers at d60. Scale bars: 100 μm in B; 200 μm in E-K″; 50 μm in L-L″.

To monitor and isolate *SOX2*-positive cells in human inner ear organoids, we engineered a hESC reporter line to express the tandem dimer Tomato (tdTomato) protein in the nuclei of *SOX2*-expressing cells (*SOX2*-2A-ntdTomato; *SOX2*^ntdTomato^) using WA25 hESCs as the parental line. We used the CRISPR-Cas9 system to insert a 2A-tdTomato-nuclear localizing signal (NLS) gene cassette at the stop codon of *SOX2* (Fig. 1A). PCR and Sanger sequencing results confirmed correct bi-allelic cassette integration at the *SOX2* stop codon locus (Fig. 1C,D). The resultant hESC line exhibited expression of the pluripotency markers OCT3/4 and SSEA4 in all *SOX2*^ntdTomato^-positive cells (Fig. S1A). Live imaging of this hESC line showed *SOX2*^ntdTomato^ expression in all undifferentiated hESCs, as expected (Fig. 1B). The reporter expression completely overlapped endogenous SOX2 (Fig. S1B,C), confirming the faithful expression of *SOX2*^ntdTomato^.

Validated *SOX2*^ntdTomato^ hESCs were differentiated into inner ear organoids using our published protocols (Koehler et al., 2017; Ueda et al., 2022; Nie et al., 2022) with modifications. Aggregated hESCs (Fig. 1E) developed otic-epibranchial placode domain (OEPD)-like epithelium on differentiation d3 (Fig. 1F,G), consistent with previous reports (Koehler et al., 2017; Nie et al., 2022); at this time, *SOX2*^ntdTomato^ was weakly expressed on the surface of the aggregates. After adding the Wnt agonist CHIR-99021 on d7, the surface structures ruffled and built otic-cyst-like structures and, by d15, organized into vesicles that exhibited stronger *SOX2*^ntdTomato^ expression (Fig. 1H). *SOX2*^ntdTomato^ expression in these vesicles was maintained until d40 of differentiation (Fig. 1I,J). Immunofluorescence showed that expression of the otic placode markers PAX2 and PAX8 colocalized with *SOX2*^ntdTomato^ expression in vesicles (Fig. 1K), consistent with our previous study (Koehler et al., 2017). *SOX2*^ntdTomato^ was also detectable in peri-vesicle regions and the aggregate core, indicating the presence of SOX2^+^ nonotic cells, such as proliferating cells, surface ectodermal cells, neural crest cells and neuroblasts (Koehler et al., 2017). By d60, MYO7A^+^ *SOX2*^ntdTomato+^ hair cells with the projection of TUJ1^+^ neuronal fibers were identified (Fig. 1L) as reported previously (Koehler et al., 2017).

### Cell type profiling of *SOX2*-expressing cells reveals multiple otic lineage populations in human inner ear organoids

**Fig. 2.**
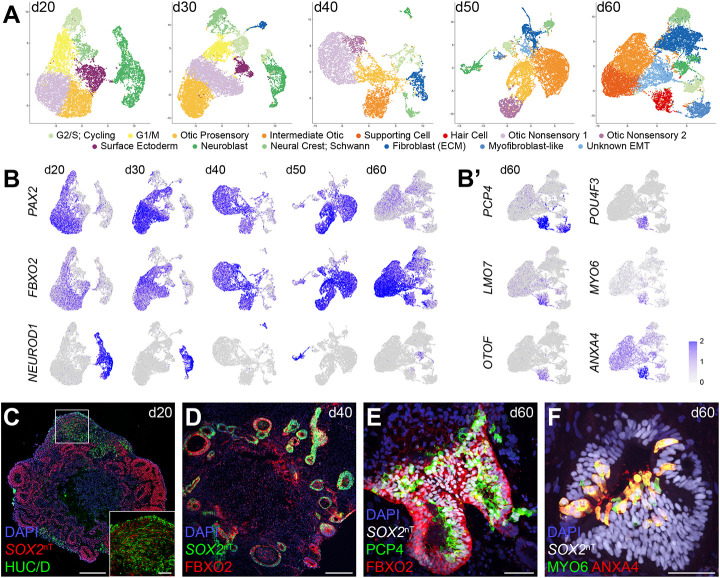
**Cell types detected in human inner ear organoids at various time points during differentiation.** (A) UMAP plot showing identified clusters in d20 (12,112 cells), d30 (14,189 cells), d40 (7521 cells), d50 (9963 cells) and d60 (17,584 cells) datasets. (B) Feature plots of representative marker expression of cycling cells (*TOP2A*), otic progenitors (*PAX2*), otic sensory epithelium cells (*FBXO2*) and otic neural progenitors (*NEUROD1*) in individual datasets. (B′) Hair cell marker expression in the d60 sample. (C-F) Immunofluorescence for the neural marker HUC/D (d20), otic progenitor markers PAX2 and FBXO2 (d40), and hair cell markers PCP4, POU4F3 and ANXA4 (d60). Scale bars: 50 μm.

To obtain single-cell RNA sequencing (scRNA-seq) data over the developmental period from otic progenitors to hair cells, *SOX2*^ntdTomato+^ cells were collected by FACS from d20-60 organoids in 10-day increments (Fig. S2) and cDNA libraries were separately prepared for each time point. Individual scRNA-seq data from the five time points revealed multiple cell types in *SOX2*^ntdTomato^-positive cells (Fig. 2 and Fig. S3A). In every dataset, otic lineage cells were defined by the following expression: early prosensory cell marker *JAG1*; otic epithelium markers *EPCAM* and *FBXO2*; supporting cell markers *BRICD5* and *SPARCL1*; and hair cell markers *PCP4*, *MYO6* and *POU4F3* (Fig. 2A,B, Figs S3A and S4A). *PAX2*^+^ immature otic and *FBXO2*^+^ intermediate otic cells were found in every dataset, whereas *FBXO2* expression became stronger as differentiation progressed, especially in supporting cell populations (Fig. 2A,B and Fig. S3A). We detected a distinct *PCP4*^+^ *LMO7*^+^ *OTOF*^+^ *POU4F3*^+^ *MYO6*^+^ *ANXA4*^+^ hair cell population in d60 samples (Fig. 2B′). Although d50 samples contained cells expressing these hair cell markers, they were not identified as a distinct cluster and were instead grouped with neuronal cells (Fig. S4B). The expression of otic markers (PAX2 and FBXO2), neural markers (HUC/D) and hair cell markers (PCP4, ANXA4, and MYO6) was verified with immunofluorescence staining (Figs 1K,L and 2C-F). *DACH1*^+^ *WNT2B*^+^ *OC90*^+^ nonsensory cells (Yamamoto et al., 2021; Choo et al., 2006; Nikki et al., 2012) were also found in our dataset. Considering the existence of DACH1^+^ cells at d30-40 and their disappearance at d50 (Fig. S5A-C), these *DACH1*^+^ *WNT2B*^+^ cells in the dataset could be nonsensory progenitors. Adjacent to this population, *PTGDS*^+^ *SPP1*^+^ *OC90*^+^ cells, another putative nonsensory population, was also found; these are presumably dark cell-like cells (Scheffer et al., 2015; Wilkerson et al., 2021), although they appeared to be immature in their *SOX2* expression in our dataset. Consistent with previous reports (Steevens et al., 2019; Gu et al., 2016), LRP2^+^ dark cell-like cells lost SOX2 expression at d40-50 (Fig. S5D,E). In the d50 and d60 datasets, we detected unknown epithelium cells expressing KRT9, KRT18, KRT19, S100A6 and TAGLN; these cells might be under epithelial-mesenchymal transition (EMT) from otic epithelial cells to periotic mesenchymal cells, such as S100B^+^ cells (Figs S5F,G and S3).

Other than the otic lineage cell types, we also detected *TOP2A*^+^ *UBE2C*^+^ *MKI67*^+^ cycling cells and *PCNA*^+^ *NASP*^+^ *TYMS*^+^ G1/S cells (Schönenberger et al., 2015; Richardson et al., 2000; Grant and Cook, 2017), as well as *NEUROD1*^+^ *ELAVL4*^+^ *POU4F1*^+^ otic neuroblasts. We found an *S100B*^+^ *PLP1*^+^ *MPZ*^+^ *SOX10*^+^ *CDH19*^+^ neural-crest-like population from d20 to d60, although this cell population could include immature Schwann cells, especially at later time points based on the overlap of marker expression (*GAP43* and *NGFR*) (Fig. S3C,D). Surface ectoderm cells (*TFAP2B*, *TFAP2A*, *WNT6*, *KRT8*, *KRT18* and *KRT19*) were found only at d20 and d30, but were missing at later time points (Fig. 2A and Fig. S3A,B), probably owing to the immature identity of this cell population. A type I collagen-rich fibroblast-like population that is *COL1A1*^+^ *COL1A2*^+^ *POSTN*^+^ (LeBleu and Neilson, 2020) was detected as a small population at d30 and increased in number at later time points (Fig. 2A and Fig. S3A). These fibroblast-like cells may contribute to forming cartilage-like tissues, especially from d40 in the culture (Fig. S1D). Additionally, *S100A6*^+^ *TPM1*^+^ *TRPM3*^+^ *ACTA2*^+^ myofibroblast-like cells (LeBleu and Neilson, 2020) were found in the d50 and d60 samples (Fig. 2A and Fig. S3A). They may differentiate into muscle tissues, which might explain occasional twitches of the aggregates observed during live imaging.

### Identification of a developmental trajectory from otic progenitors to hair cells in human inner ear organoids

**Fig. 3.**
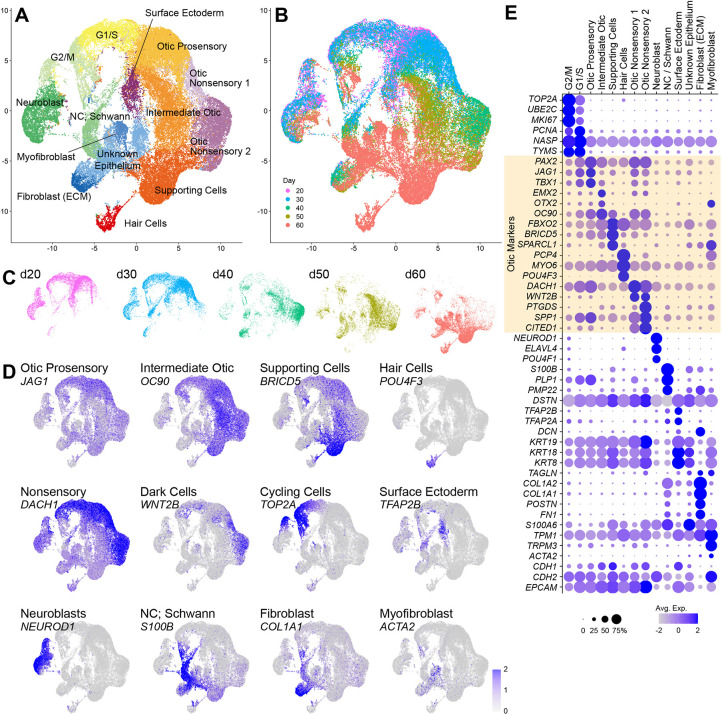
**Cell types detected in a merged dataset of d20-60 human inner ear organoids.** (A) UMAP plot showing identified clusters in the d20-60 merged dataset. (B,C) UMAP plots showing the contribution of individual sample data. (D) Feature plots showing marker expressions for otic prosensory cells (*JAG1*), intermediate otic cells (*OC90*), supporting cells (*BRICD5*), hair cells (*POU4F3*), nonsensory cells 1 (*DACH1*), nonsensory cells 2 (*DACH1* and *WNT2B*), cycling cells (*TOP2A*), surface ectoderm and neural crest cells (*TFAP2B*), inner ear neuronal progenitors (*NEUROD1*), neural crest and Schwann cells (*S100B*), fibroblast (*COL1A1*) and myofibroblast (*ACTA2*). (E) Dot plots of cycling, otic, neuronal, mesenchymal and epithelial genes from clusters shown in A.

Next, we merged the datasets for the five time-points using the merge function of the Seurat pipeline, which combines multiple datasets without transforming the data. The merged dataset with a total of 62,202 cells was processed for clustering and then the data were visualized as a UMAP plot (Fig. 3). The same set of cell clusters identified in individual time-point data was also identified in the merged dataset (Fig. 3A,E and Fig. S6). The downregulation of *PAX2* and upregulation of *FBXO2* from otic prosensory cells to intermediate otic cells and supporting cells were confirmed with this analysis (Fig. 3E). A *DACH1*^+^ *SPP1*^+^ population was detected adjacent to *OC90*^+^ intermediate otic cells (Fig. 3D, Figs S7 and S8), suggesting the existence of another lineage direction in the otic lineage other than the sensory epithelium. *TFAP2B*^+^ surface ectoderm cells, *NEUROD1*^+^ neuroblasts and *S100B*^+^ neural crest/Schwann cells were identified as distinctive clusters (Fig. 3A,D and Fig. S7). An intermediate population between otic prosensory cells and supporting cells expressed not only otic epithelium markers *EPCAM* and *FBXO2*, but also other otic progenitor markers *COL9A1*, *SERPINF1*, *HES1*, *AATF*, *COL14A1*, *OC90*, *PRSS8* and *EYA4* (Fig. S7) (Honda et al., 2017; Wilkerson et al., 2021; Sinkkonen et al., 2011; Hartman et al., 2015; Wayne et al., 2001; Tateya et al., 2011), suggesting that this is an otic epithelial cell population differentiating into hair cells and supporting cells. Adjacent to the supporting cell cluster, there was a *POU4F3*^+^ hair cell cluster that expressed vestibular hair cell markers [*CD164L2*, *ZBBX*, *TEKT1*, *SKOR1*, *TCTEX1D1* (*DYNLT5*) and *NEUROD6*] and type II hair cell markers (*ANXA4*, *MAPT* and *NHLH1*) (Fig. 3D and Fig. S8), indicative of an immature type II hair cell identity. However, we were unable to detect the cochlear marker GATA3 or INSM1 in PCP4^+^ hair cells (Fig. S8G,H). Surprisingly, we were unable to identify common progenitor populations that differentiate into both hair cells and supporting cells in the merged datasets.

**Fig. 4.**
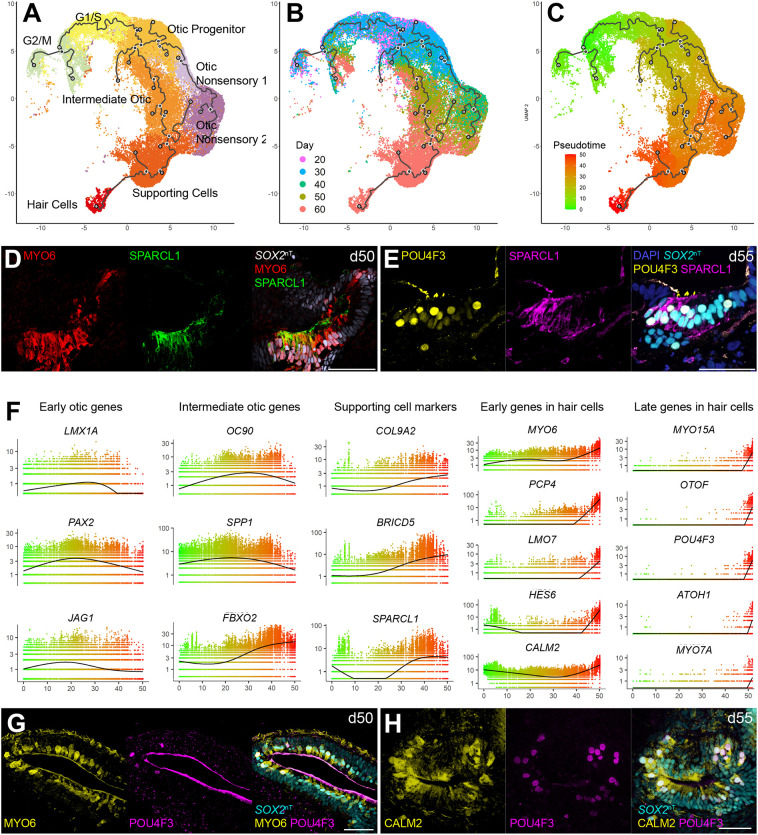
**Trajectory analysis of otic lineage cells in human inner ear organoids.** (A) Detected trajectories for cycling cells and otic lineage cells on the UMAP plot. (B) The trajectories showing the contribution of individual sample data. (C) The pseudotime from cycling cells to otic lineage cells shown on the UMAP plot. (D) MYO6^+^ SPARCL1^+^ cells in a d50 aggregate. (E) POU4F3^+^ SPARCL1^+^ cells in a d55 aggregate. (F) The expression level of otic markers along with the pseudotime. (G,H) MYO6 (G) and CALM2 (H) expression in hair cells devoid of POU4F3 expression. Scale bars: 50 μm in D,E; 50 μm in G,H.

We then selected clusters representing proliferating and otic lineage cells from the merged dataset and performed pseudotime analysis to reveal otic differentiation trajectories. These clusters included those for G1/S, G2/M, otic prosensory cells, intermediate otic cells, supporting cells, hair cells and nonsensory cells. Using the Monocle 3 pipeline (Cao et al., 2019), we found a trajectory leading from proliferating cells to hair cells via intermediate otic cells (Fig. 4A,B). The pseudotime along this trajectory was calculated using Monocle 3 by setting the point with the strongest expression of *TOP2A*, *UBE2C* and *MKI67* as the earliest time point (Figs 3D, 4C, and Fig. S7). As indicated in the developmental trajectory, we found SPARCL1^+^ MYO6^+^ cells and SPARCL1^+^ POU4F3^+^ cells in the transitional state, suggesting hair cell differentiation via supporting cells (Fig. 4D,E).

Based on this pseudotime, we tested whether temporal changes in the expression level for known otic lineage markers followed expected temporal orders (Fig. 4F). Expression of the otic progenitor markers *PAX2* and *JAG1* persisted later than that of the OEPD marker *LMX1A*. Expression of the intermediate otic markers *OC90* and *SPP1* peaked in the middle of the pseudotime plots, whereas the otic epithelium marker *FBXO2* was upregulated later than the intermediate otic markers. Expression of the supporting cell markers *COL9A2*, *BRICD5* and *SPARCL1* escalated later in the pseudotime. Based on the pseudotime plots, *MYO6*, *PCP4*, *LMO7*, *HES6* and *CALM2* were upregulated earlier than other hair-cell marker genes, such as *MYO15A*, *OTOF*, *POU4F3*, *ATOH1* and *MYO7A*, suggesting that some of these early hair cell markers we have identified in this study could act as drivers to trigger hair cell differentiation. In support of the scRNA-seq data, we found POU4F3^−^ hair cell-shaped cells expressing MYO6 or CALM2 (Fig. 4G,H) on the luminal surface of *SOX2*^+^ vesicles. The developmental trajectory exhibited linear progression from otic epithelial cells to supporting cells and from supporting cells to hair cells, and no dual trajectory branching into supporting cells and hair cells was identified in our data.

### Genes associated with functional maturation of hair cells are enriched in supporting cells

**Fig. 5.**
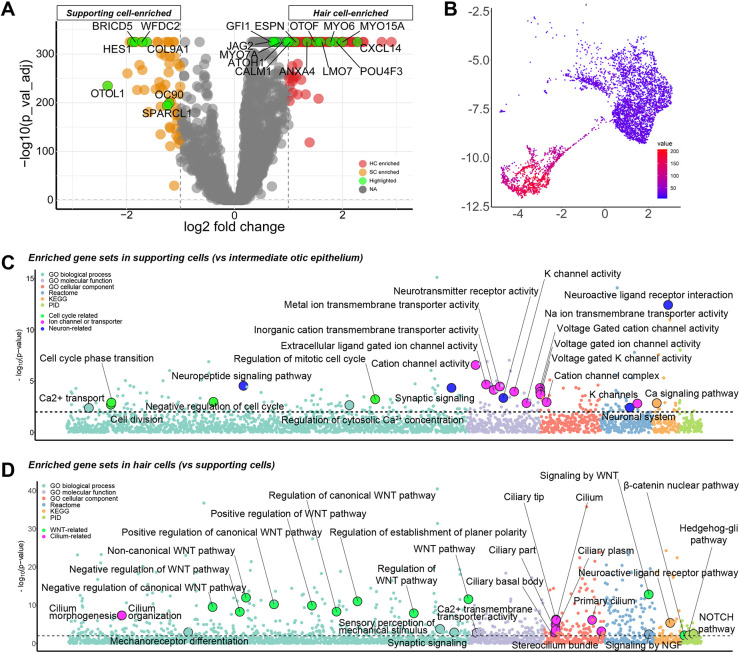
**Differentially expressed gene (DEG) analysis between the hair cell and supporting cell clusters in human inner ear organoids.** (A) Volcano plot of differentially expressed genes between hair cells and supporting cells. (B) Selected cell populations for identification of early upregulated genes in hair cells using the pseudotime analysis. (C) Bubble plot showing enriched gene sets in supporting cells in comparison with intermediate otic cells. (D) Bubble plot showing enriched gene sets in hair cells in comparison with supporting cells.

**Fig. 6.**
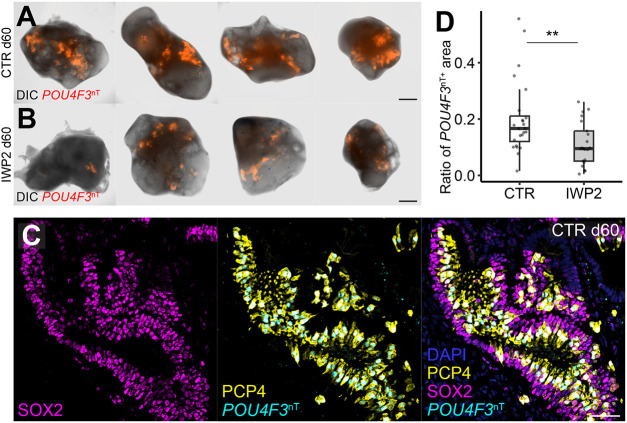
**Wnt inhibition suppresses hair cell differentiation in human inner ear organoids.** (A,B) *POU4F3*^ntdTomato+^ signals in control aggregates (A) or IWP2-treated aggregates (B). (C) Hair cells generated from *PAX2*^nEGFP^:*POU4F3*^ntdTomato^ hESCs. (D) Quantification of the ratio of *POU4F3*^ntdTomato+^ areas relative to total areas in aggregate. **P<0.01 (Wilcoxon test). Control (CTR), *n*=24; IWP2, *n*=21 from three individual batches. Scale bars: 500 μm in A,B; 50 μm in C. Boxes represent the interquartile range with the medians in the middle; whiskers show the data range.

We next asked the question of which genes are uniquely upregulated in hair cells versus supporting cells. We selected the hair cell and supporting cell clusters from the merged Seurat dataset, and created a volcano plot showing differentially expressed genes between these clusters (Fig. 5A). At the hair-cell-increasing phase, around d50-60, the expression levels of *CXCL14*, *MYO15A*, *MYO6*, *LMO7*, *POU4F3* and *OTOF* in hair cells were significantly higher than that in supporting cells. In contrast, *ATOH1*, *CALM1*, *MYO7A*, *JAG2* and *GFI1* exhibited moderately elevated expression in hair cells. Based on the trajectory found by Monocle 3, we selected cells from the branching point toward the hair cells and supporting cells (Fig. 5B and Fig. S9A) to search for gene modules that are enriched in hair cells and supporting cells, along with their trajectory. Using this selected dataset, trajectory-variable genes were collected into modules based on the strength and pattern of the expression in the selected dataset, and selected hair cell- and supporting cell-enriched modules (Fig. S9B) were plotted along with the pseudotime (Figs S10 and S11). In those early contributed genes, we found genes previously reported as hair cell marker genes (Scheffer et al., 2015), including genes encoding motor protein- or actin filament-related proteins (*EVL*, *KIFAP3*, *SMPX*, *ESPN*, *EPS8L2*, *KIF9*, *LMO7* and *PLS1*), calcium-binding proteins (*CALM1*, *CALM2*, *CETN2*, *PCP4* and *ANXA4*) and ion transporters (*ATP1B1* and *ATP6V1A*), suggesting that genes for hair cell functional maturation are upregulated before *ATOH1* expression (Figs S10, S11 and Fig. 4F) The Seurat-based data set containing hair cells and supporting cells was also processed in the DESeq2 pipeline (Love et al., 2014) followed by Gene Set Enrichment Analysis (GSEA) of upregulated genes in hair cells and supporting cells using the iDEA pipeline (Ma et al., 2020). Gene Ontology (GO) gene sets associated with ion channel- and ion transmitter-related processes, as well as neuronal gene sets, were upregulated in supporting cells in comparison with immature otic epithelial cells (Fig. 5C). Gene sets related to Wnt signaling and stereocilium development were found to be upregulated in the hair cell population (Fig. 5D), whereas those related to proliferation-, differentiation- and muscle fiber-related processes were found to be enriched in the supporting cell population (Fig. S9C). This suggests that Wnt signaling could be involved in the initiation phase of hair cell differentiation. To test this hypothesis, we grew *POU4F3*^ntdTomato^ hESC-derived organoids (Moore et al., 2022 preprint) in the presence or absence of the potent Wnt inhibitor IWP2, starting at d30. We identified *POU4F3*^ntdTomato+^ puncta in both control and IWP2-treated aggregates (Fig. 6A,B), and confirmed that these *POU4F3*^ntdTomato+^ cells also express ANXA4^+^ and SOX2^+^ (Fig. 6C). The ratio of *POU4F3*^ntdTomato+^ areas relative to the entire aggregate area was significantly lower in the IWP2-treated group than in the control (*W*=370; *P*=0.0066; Fig. 6D). These data strongly suggest that Wnt signaling plays an important role for hair cell development in the human inner ear organoid system, as reported *in vivo* (Dabdoub and Kelley, 2005; Shi et al., 2012; Ellis et al., 2019; Jacques et al., 2012).

## DISCUSSION

In the murine inner ear, Sox2 expression defines prosensory cells that give rise to both mechanosensitive hair cells and supporting cells (Dabdoub et al., 2008; Kempfle et al., 2016; Steevens et al., 2019). To identify genes that are essential for hair cell differentiation from prosensory cells, we studied temporal changes in the transcriptome of *SOX2*^+^ cells isolated from human inner ear organoids that exclusively generate vestibular hair cells (Koehler et al., 2017). Although SOX2 expression declines in type I vestibular hair cells and cochlear hair cells as they mature (Dabdoub et al., 2008; Lu et al., 2019; Stone et al., 2021), its expression is retained in supporting cells and type II vestibular hair cells – the primary hair cell type in human inner ear organoids (Koehler et al., 2017). By taking advantage of the persistent expression of SOX2 in these cell populations, we were able to collect scRNA-seq data for *SOX2*-expressing cells at developmental stages ranging from multipotent otic progenitors to terminally differentiated hair cells and supporting cells. Multiple otic lineage cells were identified in samples at every time point from d20 to d60, and the merged dataset of each time point revealed differentiating cell types, as well as cycling cells, otic progenitors, supporting cells and hair cells in a linear developmental trajectory. Contrary to the current consensus in the field, however, we were unable to find common bipotential prosensory cells that can differentiate into either hair cells or supporting cells. Our psudotime data instead suggest that hair cells arise from some of the supporting cell populations in human inner ear organoids. Although transdifferentiation from supporting cells to hair cells appears to take place during regeneration (Wang et al., 2015; Benkafadar et al., 2021; Bramhall et al., 2014; Shu et al., 2019), we did not observe upregulation of the dying hair cell markers *IFIH1*, *XPA*, *ATP4B*, *BRCA2*, *MYT1*, *PCF11* and *TRIM35* (Benkafadar et al., 2021) in the hair cell cluster (Fig. S12A) or upregulation of cleaved caspase 3 (*CC3*) in hair cells (Fig. S12C,D). Moreover, the proliferation marker phosphorylated histone H3 (pH3) was undetectable in both hair cells and supporting cells (Fig. S12B). These results exclude the possibility of active hair cell regeneration in our samples. One factor that could have potentially contributed to this seemingly contradictory result is the immaturity of hair cells in our samples. The number of hair cells increases even after d60 in organoid culture and late-born hair cells were not included in our analysis. Additionally, hair cells in our samples did not exhibit strong expression of hair-cell marker genes such as *ATOH1*, *POU4F3*, *MYO7A* and *GFI1* (Fig. 5A). It should be noted, however, that previous studies reported evidence for hair cell differentiation via intermediate supporting cells in chicks (Jan et al., 2021) or supporting cell-like progenitors in mice (Burns et al., 2015). The question of whether dual-fated common progenitors exist in our system and, if so, how progenitors and supporting cells contribute to hair cell differentiation awaits further investigation using more-mature organoid samples.

In contrast to the immature state of hair cells, supporting cells in d50 and 60 samples seemed to be relatively mature as they strongly expressed well-known supporting-cell marker genes, such as *HES1*, *COL9A2*, *BRICD5* and *SPARCL1* (Fig. 3E, Figs S3A, S4A, S5F,G, and S13). These supporting cells expressed extrastriolar-enriched genes of postnatal mice (*OC90*, *DKK3*, *ALDH1A1*, *ALDH1A3* and *CRYAB*) more strongly than striolar-enriched genes (*TECTB*, *CYP26B1*, *CPA6*, *ISL1*, *SFRP2*, *RCAN1* and *BAMBI*) (Fig. S13) (Jan et al., 2021). This, along with the lack of crista-specific supporting cell gene expression (*ZPLD1*, *AGR3* and *MEIS2*) (Fig. S13) (Wilkerson et al., 2021), suggests the identity of the detected population in this study as maturing extrastriolar supporting cells. Our GSEA revealed significantly higher expression of ion channel- and ion transporter-related gene sets in supporting cells compared with intermediate otic cells (Fig. 5D), suggesting functional maturation of supporting cells as excitable cells. Furthermore, the trajectory in the subclusters of supporting cells was continuous from the BRICD5^+^ SPARCL1^+^ population (Fig. S14). Moreover, we found cells in SOX2-positive vesicles expressing both supporting cell and hair cell markers (Fig. 4D). Thus, relatively mature supporting cells in organoids appeared to be competent to give rise to hair cells in our system. These competent supporting cells appear to be responsive to Wnt signaling when differentiating into hair cells (Fig. 6), consistent with previous reports that Wnt activation promotes hair cell differentiation from mature supporting cells *in vivo* (Jacques et al., 2012; Shi et al., 2013).

During early otic development, SOX2^+^ JAG1^+^ prosensory cells build up in otocysts and later give rise to hair cells and supporting cells, which is accompanied by upregulation of P27^KIP1^ (*CDKN1B*), NGFR (*CD271*), HEY2 and PROX1, and downregulation of JAG1 in the mouse cochlea (Dabdoub et al., 2008; Zak and Daudet, 2021; Roccio et al., 2018; Basch et al., 2011; Pan et al., 2013; Ohyama et al., 2010). Based on the expression of *SOX2*^ntdTomato^ and *JAG1*, we denoted the earliest otic lineage cells in the dataset as prosensory cells; however, we found a developmental trajectory from the prosensory cells towards nonsensory cells that express *WNT2A*, *DACH1* and *PTGDS* (Fig. 3D,E). Within the prosensory cells in the dataset, we also found a subpopulation expressing *TBX1*, a marker of the sensory and nonsensory domains in otic vesicles (Macchiarulo and Morrow, 2017), and *LMX1A*, which antagonizes NOTCH signaling and contributes to forming nonsensory cells (Brown et al., 2020) (Figs S3 and S6). These data suggest that prosensory cells denoted in the merged dataset also contain a certain number of nonsensory progenitors.

We found an intermediate otic-lineage cell population filling the gap between prosensory cells and supporting cell populations (Fig. 3A). This population expresses the known otic lineage markers *COL9A1*, *SERPINF1*, *HES1*, *AATF*, *COL14A1*, *OC90*, *PRSS8* and *EYA4* (Fig. S7) (Honda et al., 2017; Wilkerson et al., 2021; Sinkkonen et al., 2011; Hartman et al., 2015; Wayne et al., 2001; Tateya et al., 2011). In the merged dataset, the expression levels of the sensory epithelium marker *FBXO2* and supporting cell marker *SPARCL1* were unchanged from otic prosensory cells to supporting cells, while the otic progenitor markers *PAX2*, *JAG1* and *TBX1* were downregulated in intermediate otic cells compared with prosensory cells (Fig. 3D,E). These results suggest that the intermediate otic population found in this study is transitional from prosensory cells to supporting cells. A subpopulation of this cluster expresses *OTX2*, *LRP2* and *CNMD* (Fig. 3E and Fig. S7). *OTX2* expression is temporarily observed in a region between macula sacculi and macula utriculi during mouse vestibular development (Sanchez-Calderon et al., 2004). *LRP2* and *CNMD* are expressed in a nonsensory epithelium containing dark cells, adjacent to the mouse vestibular epithelium (Wilkerson et al., 2021; Jan et al., 2021). Furthermore, our trajectory analysis has revealed a sub-trajectory from the intermediate otic cell cluster to the dark cell-like nonsensory cells (Fig. 4A). These results suggest that the intermediate otic cell cluster found in this study is an amalgam of prospective supporting cells and immature nonsensory cells in the utricle and saccule.

It is important to note several limitations inherent to our current approach. First, we set the sampling endpoint at d60 as we focused on differentiation of prosensory cell populations; however, we have found that hair cell development continues well beyond d60 (Nie et al., 2022; Moore et al., 2022 preprint). This could have potentially excluded data on late-born or late-differentiating hair cells. Second, we analyzed only *SOX2*^+^ cell populations in organoid samples; this could have inevitably excluded from analysis some terminally differentiated cell types other than hair cells. For example, we were not able to perform a trajectory analysis for dark cells, which lose SOX2 expression after maturation. Third, it is possible that a small population of immature type I-fated hair cells exists in our organoid samples, as vestibular type I hair cells arise with our current differentiation protocol (Liu et al., 2016). Further modification of the protocol will be necessary to investigate the type I hair cell development in human inner ear organoids. Finally, we performed our analyses with organoid samples derived from only one hESC line. Whether the genetic backgrounds of human pluripotent stem cells have any effects on cell lineage specification awaits further investigation.

In conclusion, our scRNA-seq analysis of *SOX2*-expressing cells in human inner ear organoids at different developmental stages has identified a developmental trajectory from amplifying otic progenitors through prosensory cells, intermediate otic cells and supporting cells to hair cells. This, along with another finding in this study, that gene sets associated with ion channel- and ion transporter-related processes are enriched in supporting cells versus prosensory cells, raises the intriguing possibility that a subpopulation of supporting cells might constitute precursors of hair cells in the human inner ear. These findings provide valuable insights into how prosensory cells give rise to hair cells and supporting cells during human inner ear development, and may provide a clue to promote hair cell regeneration from resident supporting cell populations in individuals with profound hearing loss or balance disorders.

## MATERIALS AND METHODS

### Generation of a *SOX2*-2A-ntdTomato reporter hESC line

A guide RNA (gRNA, 5′-CGGCCCTCACATGTGTGAGA-3′; Integrated DNA Technologies) was designed to target the stop codon region of *SOX2* (Fig. 1A). To construct the donor vector, a 2A-tdTomato-nucleus localizing signal (NLS)-bovine growth hormone (bGH) polyA cassette [assembled from synthetic gBlocks DNA (IDT)] flanked by two 1 kb SOX2 locus homology arms PCR amplified from hESC (WA25, WiCell) genomic DNA was cloned into a pUC19 backbone. The guide RNA, the donor vector and HiFi Cas9 Nuclease V3 (Integrated DNA Technologies #1081060), as well as a vector expressing PGK-puro (Addgene 31938) (Hockemeyer et al., 2011), were transfected into WA25 hESCs with a 4D Nucleofector (Lonza) using the P3 Primary Cell 4D-Nucleofector X kit (Lonza, V4XP-3012) and Program CB-150. After nucleofection, the cells were plated with E8 flex medium (Gibco, A2858501) containing 100 μg/ml Normocin (InvivoGen, ant-nr-1) (E8fn), 1× RevitaCell (Gibco, A26445-01) and 1 μM Scr7. At 24 h post-nucleofection, the medium was changed into E8fn containing 1 μM of Scr7 and 0.5 μg/ml puromycin. At 48 h post-nucleofection, the medium was changed to E8fn medium without supplements. Clonal cell lines were established by low-density seeding (1-3 cells/cm^2^) of dissociated single hESCs followed by isolation of hESC colonies after 7-8 days of expansion. Successfully transfected colonies expressing *SOX2*-ntdTomato were picked up under a fluorescent microscope and seeded into 24-well plates followed by expansion. Genotypes of the clonal cell lines were analyzed by PCR (primers are listed in Table S1) followed by gel electrophoresis and by Sanger sequencing (ACGT) of total PCR amplicons. hESC lines with bi-allelic 2A-ntdTomato integration were used for subsequent experiments. Pluripotency of these hESC lines was assessed with the pluripotent stem-cell markers, SOX2, OCT4 and SSEA-1 (the antibodies are listed in Table S2). Routine karyotyping was not performed; however, the passage number was minimized to reduce the risk of spontaneous chromosomal duplications or genetic mutations.

### hESC culture

*SOX2*-2A-ntdTomato hESCs (passages 35-50) or *PAX2*-2A-nEGFP:*POU4F3*-2A-ntdTomato hESCs (passages 40-50) (Moore et al., 2022 preprint) were cultured in E8fn medium on Vitronectin (Invitrogen, A14700)-coated six-well plates (Thermo Fisher Scientific, 140675) according to an established protocol (Beers et al., 2012). The cells with 60-70% confluency were passaged every 3 or 4 days at a split ratio of 1:10-1:50 using 0.5 mM ethylenediaminetetraacetic acid (EDTA, Sigma-Aldrich, 93283-100ML) in Dulbecco's phosphate-buffered saline (DPBS Gibco, 14190250). The cells were incubated with E8fn supplemented with 1×RevitaCell supplement for 16-24 h and then replaced with E8fn without RevitaCell. The medium was changed every 2-3 days. All cultures were incubated at 37°C under 5% CO_2_ with 100% humidity.

### Inner ear organoid culture

To start differentiation, hESCs were dissociated with Accutase (Gibco, A1110501) and distributed, 3500 cells per well, onto low-adhesion 96-well U-bottom plates (Thermo Fisher Scientific, 12-566-430) in 100 μl of E8fn containing 20 μM Y-27632 (STEMCELL Technologies, 72304). Four hours after seeding, 100 μl of E8fn was added to reduce the concentration of Y-27632. After a 48 h incubation (i.e. differentiation day 0), the aggregates were transferred to a new low-adhesion 96-well U-bottomed plate in 100 μl of E6 medium (Gibco, A1516401) containing 4 ng/ml FGF-2 (STEMCELL Technologies, 78003), 10 μM SB-431542 (Reprocell, 04-0010-10) and 500 pg/ml BMP-4 (Reprocell, 03-0007) and 2% growth factor-reduced Matrigel (Corning, 354230). On d3, 25 μl of E6 containing 50 ng/ml FGF-2 and 200 nM LDN-193189 (Reprocell, 04-0074-02) was added in each well. On d7, the aggregates were re-transferred into a 96-well U-bottomed plate with E6 medium containing 40 ng/ml FGF-2, 200 nM LDN and 3 μM CHIR-99021. On d8, the medium was partially changed with the same medium as d7. On d11, aggregates were washed and transferred into a 100-mm dish (Corning, 353003) with Organoid Maturation Medium (OMM) containing a 50:50 mixture of Advanced DMEM:F12 (Gibco, 12-634-028) and Neurobasal Medium (Gibco, 21-103-049) supplemented with 0.5×N2 Supplement (Gibco, 17-502-048), 0.5×B27 without Vitamin A (Gibco, 12587010), 1×GlutaMAX (Gibco, 35-050-061), 0.1 mM β-mercaptoethanol (Gibco, 21985023), and 1×100 Normocin supplemented with 1% Matrigel and 3 μM CHIR-99021. The medium was changed with OMM supplemented with 3 μM CHIR-99021 on d13 and d15. After changing the medium with OMM on d18, the medium was changed with OMM twice per week. Aggregates were transferred into new dishes every 1-3 weeks. The culture was maintained up to d60. For IWP2 treatment, OMM containing 3 μM IWP2 (Tocris, 3533) was fed from d30 and maintained up to d60. A Nikon SMZ18 fluorescent stereomicroscope was used for live imaging. For imaging, a side that displayed the broadest and strongest expression of *POU4F3*^ntdTomato^ in each aggregate was selected under a Nikon SMZ18 fluorescent stereomicroscope. General Analysis 3 built-in software on NIS Elements Advanced Research application (Nikon) was used for the automatic measurement of the proportion of *POU4F3*^ntdTomato+^ areas in aggregates using the same threshold for *POU4F3*^ntdTomato+^ signals between the groups. The data were collected from 24 control aggregates and 21 IWP2-treated aggregates from three individual batches.

### Immunofluorescence

Undifferentiated hESCs were fixed with 4% paraformaldehyde (PFA, Electron Microscopy Sciences) for 15 min at room temperature, whereas aggregate samples were fixed with 4% PFA for 30 min at room temperature followed by cryoprotection with 30% sucrose. The frozen samples embedded in a tissue-freezing medium (General Data Healthcare) were sectioned at 12 μm and collected on slide glasses (Thermo Fisher Scientific). Ten percent horse serum (Vector Laboratories) in 1× PBS with 0.1% Triton -X100 (T-PBS) was used for blocking. T-PBS with 3% horse serum was used to dilute primary and secondary antibodies (Table S2). The samples were incubated with primary antibodies overnight at 4°C, followed by washing with 1×PBS and incubation with secondary antibodies for 1 h at room temperature. After washing the slides, ProLong Gold Antifade Mountant with DAPI (Invitrogen) was used to seal the stained samples with coverslips (Corning). A Nikon ECLIPSE TE2000-U inverted fluorescent microscope was used for live imaging, and a Leica DMi8 inverted fluorescent microscope and Nikon A1R HD25 confocal microscope were used for stained samples.

### Whole-mount immunofluorescence

The AbScale tissue-clearing protocol (Hama et al., 2015, 2016) was used for whole-mount immunofluorescence with modifications. Incubation and washing steps were performed on a rotor and incubation steps were performed at 37°C on a rotor unless otherwise noted. Day 20 (d20) and d60 samples were fixed with 4% (v/v) paraformaldehyde (PFA, Electron Microscopy Sciences) overnight. The samples were incubated for 6 h in Scale S0 [20% (w/v) D-(-)-sorbitol (Sigma-Aldrich), 5% (v/v) glycerol (Sigma-Aldrich), 1 mM methyl-β-cyclodextrin (Santa Cruz Biotechnology), 1 mM γ-cyclodextrin (Sigma-Aldrich), 1% (w/v) N-acetyl-L-hydroxyproline (Sigma-Aldrich), 3% (v/v) dimethylsulfoxide (DMSO; Sigma-Aldrich) and 0.1 M PBS(−) (Sigma-Aldrich); pH was adjusted to 7.2 with 0.1 M sodium hydroxide]. The samples were incubated sequentially as follows: incubation in Scale A2 [10% (v/v) glycerol, 4 M urea (Sigma-Aldrich), and 0.1% (v/v) Triton X-100 (Sigma-Aldrich)] for 16 h, in ScaleB4(0) [8 M urea] for 24 h and in ScaleA2 for 8 h. Thereafter, the samples were immersed in 0.1 M PBS(−) for 4 h at room temperature and blocked with 10% (v/v) normal horse serum (NHS, Vector Laboratories) in AbScale solution [0.33 M urea and 0.25% (v/v) Triton X-100, and 0.1 M PBS(−)] for 16 h, followed by incubation with primary antibodies diluted in AbScale solution (at the same concentration as shown above) containing 3% (v/v) NHS for 48 h. After sequential washing with the AbScale solution at room temperature for 15, 30, 60 and 120 min, the samples were incubated with fluorophore-labeled secondary antibodies diluted in AbScale solution (1:500) containing 3% (v/v) NHS for 24 h. After washing with AbScale at room temperature for 30 min and rinsing twice with AbScale Rinse solution [2.5% (w/v) bovine serum albumin (Sigma-Aldrich) and 0.05% (v/v) Tween-20 (Sigma-Aldrich), and 0.1 M PBS(−)] at room temperature for 30 min, the samples were re-fixed with 4% (v/v) PFA in 0.1 M PBS(−) at room temperature for 1 h. After washing with 0.1 M PBS(−), the samples were incubated in ScaleS4 [40% (w/v) D-(-)-sorbitol, 10% (v/v) glycerol, 4 M Urea, 0.2% (v/v) Triton X-100 and 20% (v/v) DMSO] for 16 h. On cover glasses (0.16-0.19 mm thickness, Corning) mounted with silicone gaskets (EMS and Thermo Fisher Scientific), the samples were embedded with ScaleS4 containing 2% agarose (Invitrogen). The embedded samples were left at 4°C at least overnight to immobilize. A Nikon A1R HD25 confocal microscope was used for imaging the cleared samples.

### Sample preparation for scRNA-seq

D20 and d30 aggregates were washed with 0.5 mM EDTA in DPBS, followed by incubation with 1.1 mM EDTA and TrypLE (Life Technologies, A1217701) in DPBS at 37°C for 60 min. During incubation, the samples were mechanically dissociated on Nunclon Sphera 24-well plates (Thermo Fisher Scientific, 174930) and gently pipetted with P1000 tips. After confirming that most *SOX2*^ntdTomato^-positive cells had become single cells, they were filtered sequentially through a 100 µm and a 40 µm cell strainer (Corning, 352360 and 431750, respectively) and then transferred into 2 ml tubes (Eppendorf, 022363352). After spinning down at 100 ***g*** for 5 min, the cells were resuspended with fluorescence-activated cell sorting (FACS) buffer, which consists of 2% fetal bovine serum (Gibco, A3160401) in 1× DPBS and collected into a 5 ml round-bottomed polypropylene tube (Falcon, 352063).

D40, d50 and d60 aggregates were dissected to remove cartilage-like tissues and obvious non-epithelial tissues in OMM in a 60 mm dish (Falcon, 353002) with Style 5 tweezers (Electron Microscopy Sciences, 72700D) under a Nikon SMZ18 fluorescence stereomicroscope. The dissected aggregates were kept in OMM on low cell-binding six-well plates (Corning, 3471) until dissociation. After dissociation with Accutase, *SOX2*^ntdTomato^-positive populations were collected with BD SORP Aria or BD FACSAria Fusion (BD Biosciences). Single-cell suspension after FACS was centrifuged and resuspended with FACS buffer. The cell suspension was counted with a hemocytometer under a microscope for cell number and cell viability (>85%). The single-cell gene expression analysis was conducted using a 10X Chromium single-cell system (10X Genomics). Approximately 10,000 cells per sample were loaded on a single cell master mix with lysis buffer and reverse transcription reagents, according to the Chromium Single Cell 3′ Reagent Kits V3 User Guide, CG000183 Rev A (10X Genomics). Along with the single-cell gel beads and partitioning oil, the single-cell master mixture containing the single-cell suspension was dispensed onto a Single Cell Chip B in separate wells, and the chip was loaded to the Chromium Controller for gel bead-in emulsion (GEM) generation and barcoding, followed by cDNA synthesis and library preparation. At each step, the quality of cDNA and library was examined by Bioanalyzer and Qubit. The resulting library was sequenced in a custom program for 28b plus 91b paired-end sequencing on an Illumina NovaSeq 6000 sequencer. Over 27K reads per cell were generated and 93% of the sequencing reads reached Q30 (99.9% base call accuracy).

### scRNA-seq data analysis

Illumina's Real-Time Analysis software was used to generate a BCL file, which was subsequently de-multiplexed and converted to a FASTQ file using the bcl2fastq Conversion Software (Illumina). The Cell Ranger pipeline was used to process the FASTQ file as follows. De-multiplexed reads were mapped to the GRCh38/hg38 human reference genome with the STAR (Spliced Transcripts Alignment to a Reference) aligner, mapped reads were grouped by cell barcode and single-cell gene expression was quantified using unique molecular identifiers (UMIs). The resulting filtered gene-barcode (count) matrix was used as input for downstream analysis.

RStudio v2021.09.0 running R language v4.1.2 was used as a platform for all analysis of our scRNA-seq data. The initial normalization and clustering were performed by Seurat v4.1.0. (Hao et al., 2021). The matrix of read-count data of each cell for each gene was loaded individually and converted to Seurat objects. Cells containing extremely high or low UMI counts or an extremely high percentage of mitochondrial gene counts were removed from the pool. The gene expression levels were normalized by SCTransform function and variable gene identification was carried out at the same time. Dimension reduction was performed by principal component analysis (PCA) and the number of the principal components was determined based on the elbow plot of the standard deviation explained by each principal component. Shared nearest neighbor graph construction and unsupervised clustering were performed, followed by finding differentially expressed genes (DEG) in each cluster for determination of cluster identity, and a uniform manifold approximation and projection plot (UMAP) was calculated. For the merged data, after filtering out low-quality data, each dataset of d20, d30, d40, d50 and d60 after SCTransform was merged into one dataset, followed by the same process for each dataset. The DEGs in the supporting cell and hair cell clusters found by Seurat were used for the volcano plot. We used a 0-2 scale for the expression level in the future plots unless otherwise indicated.

The expression matrix stored in the Seurat dataset was inherited by the CellDataSet class of Monocle 3 v1.0.0 (Cao et al., 2019) along with the information pertaining to dimensional reduction, clustering and the UMAP generated in Seurat. After preprocessing and clustering, the developmental trajectory was calculated and then pseudotime was calculated by setting the starting point of differentiation based on the expression of the cell-cycle genes, *TOP2A*, *UBE2C* and *MKI67*. For detection of the developmental trajectories in the supporting cell sub-clusters and hair cells, we selected the supporting cells and hair cells from the merged dataset and performed reclustering by the Seurat pipeline, and then passed them to the Monocle pipelines. For the pseudotime analysis, after selecting a cell subset in the branch point between the supporting cell and hair cells according to the result of choose_graph_segments function (Fig. S9A), every gene was sorted into expression modules with a resolution that allowed the detection of highly expressed genes in hair cells in one module (Fig. S9B). The genes highly expressed in hair cells were selected for pseudotime plots to find early upregulated genes during hair cell differentiation.

The hair cells, supporting cells and intermediate otic clusters were processed by DESeq2 v1.34.0 (Love et al., 2014), followed by Gene Set Enrichment Analysis (GSEA) using the Integrative Differential expression and gene set Enrichment Analysis (iDEA) v1.0.1. Gene sets used for GSEA were downloaded from the Molecular Signatures Database (MSigDB) (http://www.gsea-msigdb.org/gsea/msigdb/collections.jsp). Entire lists of enriched gene sets revealed by the GSEA analysis are shown in Table S3. All source codes were uploaded to the GitHub repository (https://github.com/HashinoLab/Ueda_et_al_SOX2_tdTomato). DESeq2 and GSEA codes were run on high-throughput computing cluster and storage resources at Indiana University.

### Statistics

RStudio running R language was used for statistical analysis for IWP2 treatment data. The Wilcoxon signed-rank test was carried out for the comparison of *POU4F3*^ntdTomato+^ areas between control and IWP2-treated groups.

## Supplementary Material

10.1242/develop.201071_sup1 Supplementary information
