# Supplementary material for: Defining developmental trajectories of prosensory cells in human inner ear organoids at single-cell resolution
Source: Development. 2023 Jun 29;150(12):dev201071. doi: 10.1242/dev.201071 (PMC10323240; doi:10.1242/dev.201071)
Supplement: Supplementary information [file develop-150-201071-s1.pdf]

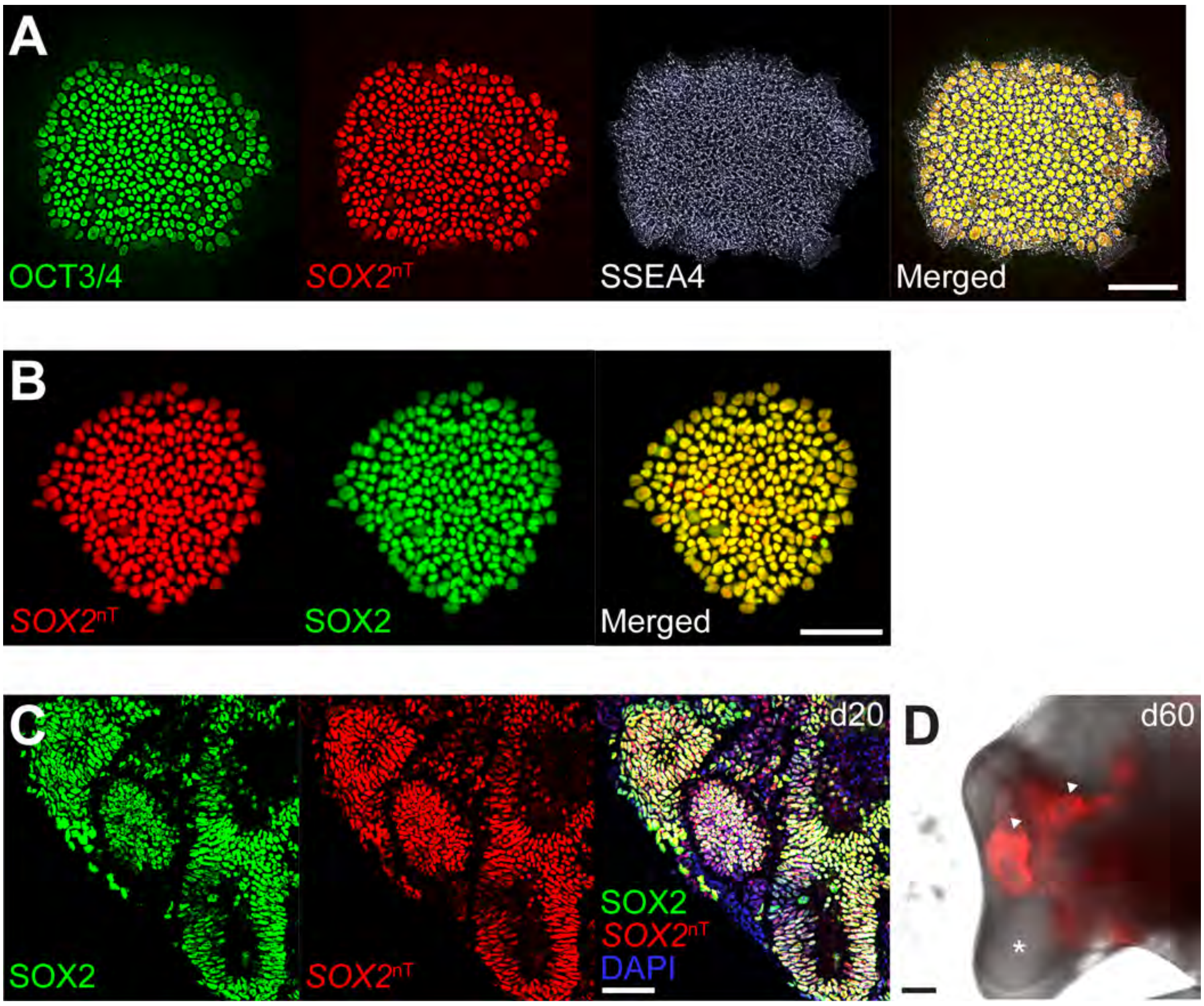

Fig. S1. Validation of the *SOX2*-2A-ntdTomato hESC line.

(A) Pluripotency marker (OCT3/4 and SSEA4) and *SOX2*^{ntdTomato} expression in undifferentiated hESCs. (B) Colocalization of *SOX2*^{ntdTomato} reporter expression and SOX2 protein expression in undifferentiated hESCs. (C) Colocalization of *SOX2*^{ntdTomato} reporter and SOX2 protein expression in a d20 inner ear organoid. (D) *SOX2*^{ntdTomato} expressing vesicles (arrowheads), adjacent to cartilage-like tissues (asterisk). Scale bars: A and B, 100 µm; C and D, 200 µm

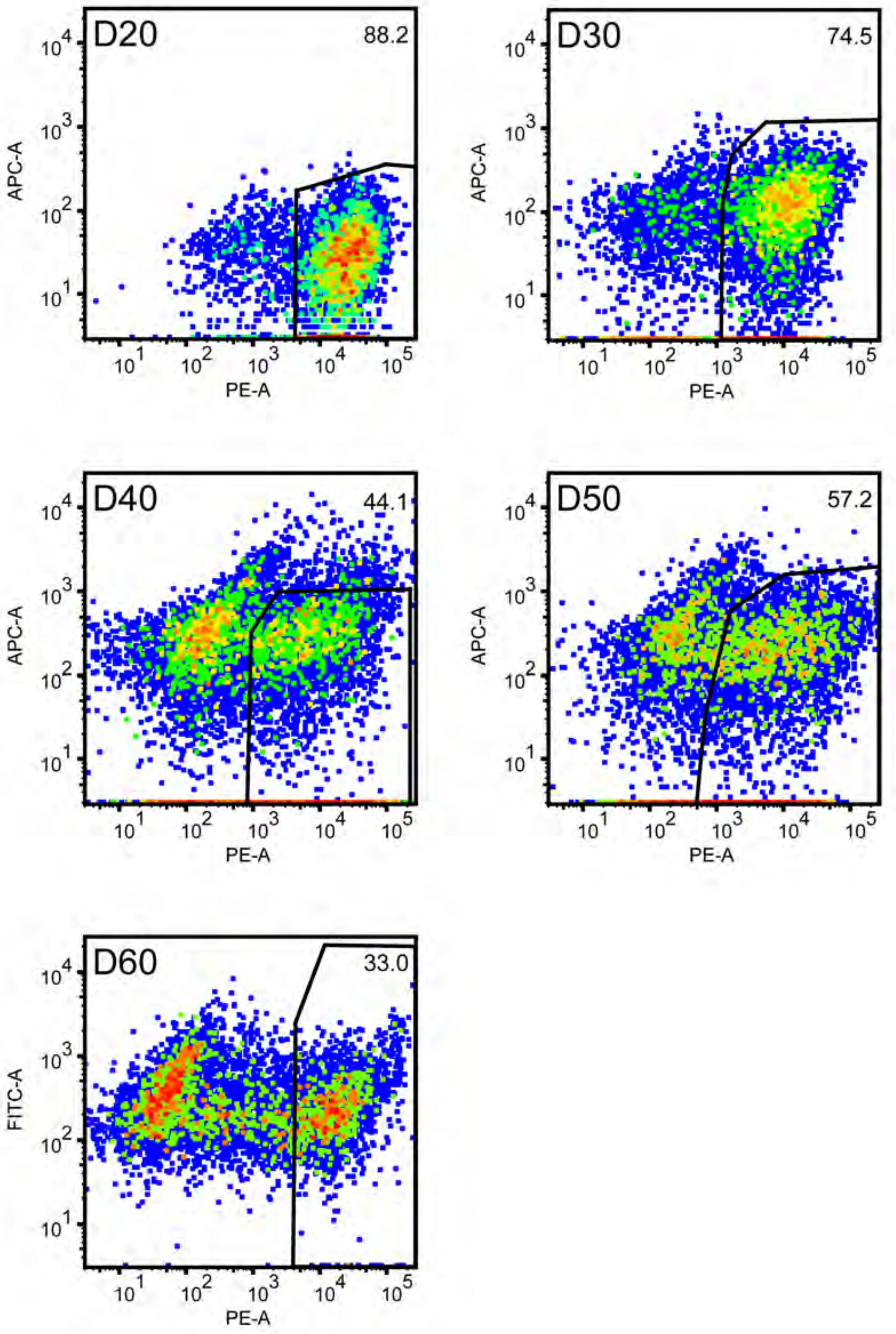

Fig. S2. FACS gating strategy to isolate *SOX2*-positive cells from whole aggregates.

FACS isolation of *SOX2*^{ntdTomato}-positive cells from d20, d30, d40, d50, and d60 inner ear organoids.

Development • Supplementary information

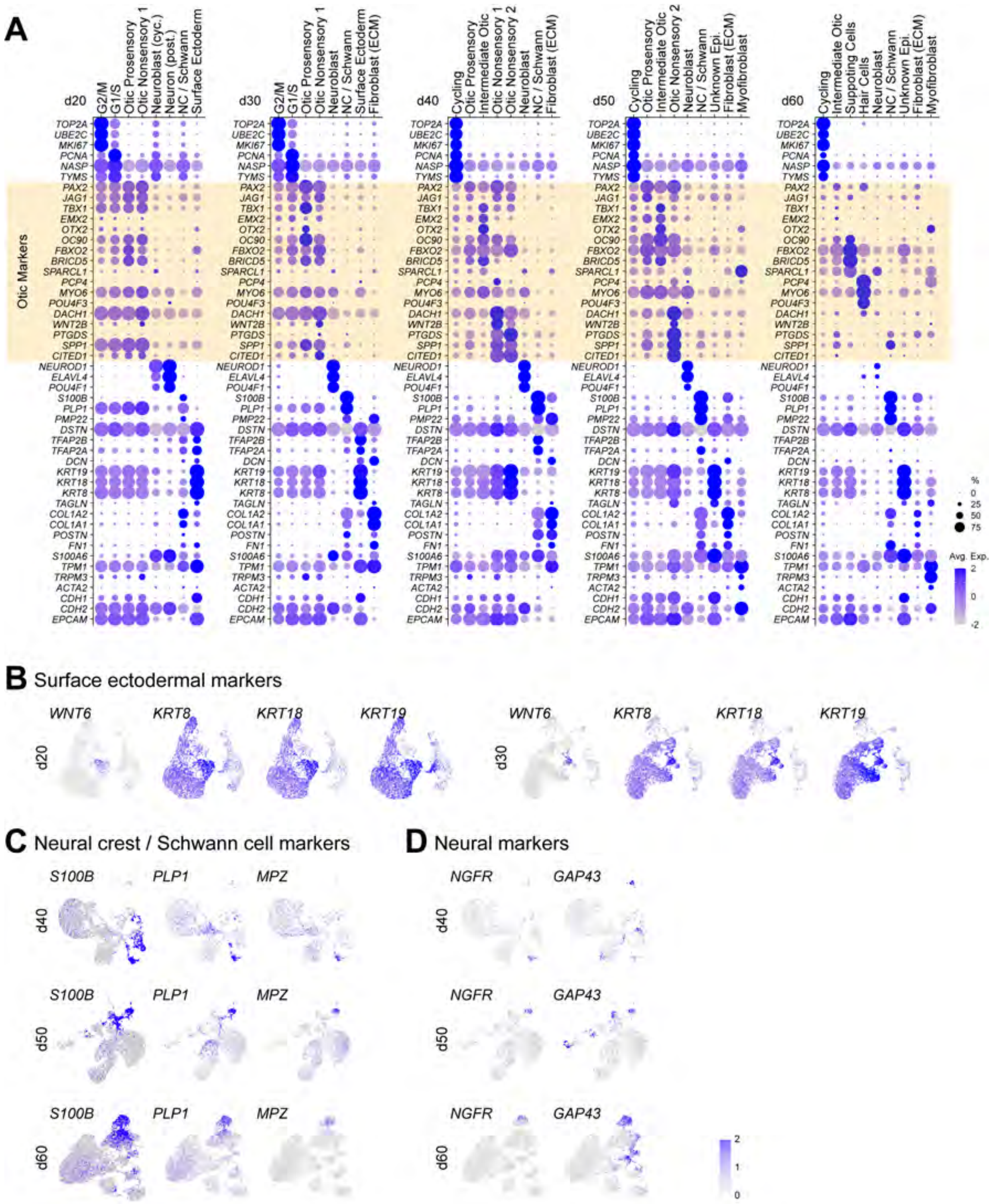

Fig. S3. Dot plots and feature plots for ectodermal and neural/glial markers.

(A) Dot plots for cycling, otic, neuronal, mesenchymal, and epithelial genes from clusters shown in Fig. 2A. (B) Feature plots of d20 and d30 datasets showing the expression of surface ectodermal markers (*WNT6*, *KRT8*, *KRT18*, and *KRT19*). (C) Feature plots of d40-60 datasets showing neural crest and Schwann cell markers (*S100B*, *PLP1*, *MPZ)*, and neural markers expressed in Schwann cells (*NGFR* and *GAP43)*.

Development • Supplementary information

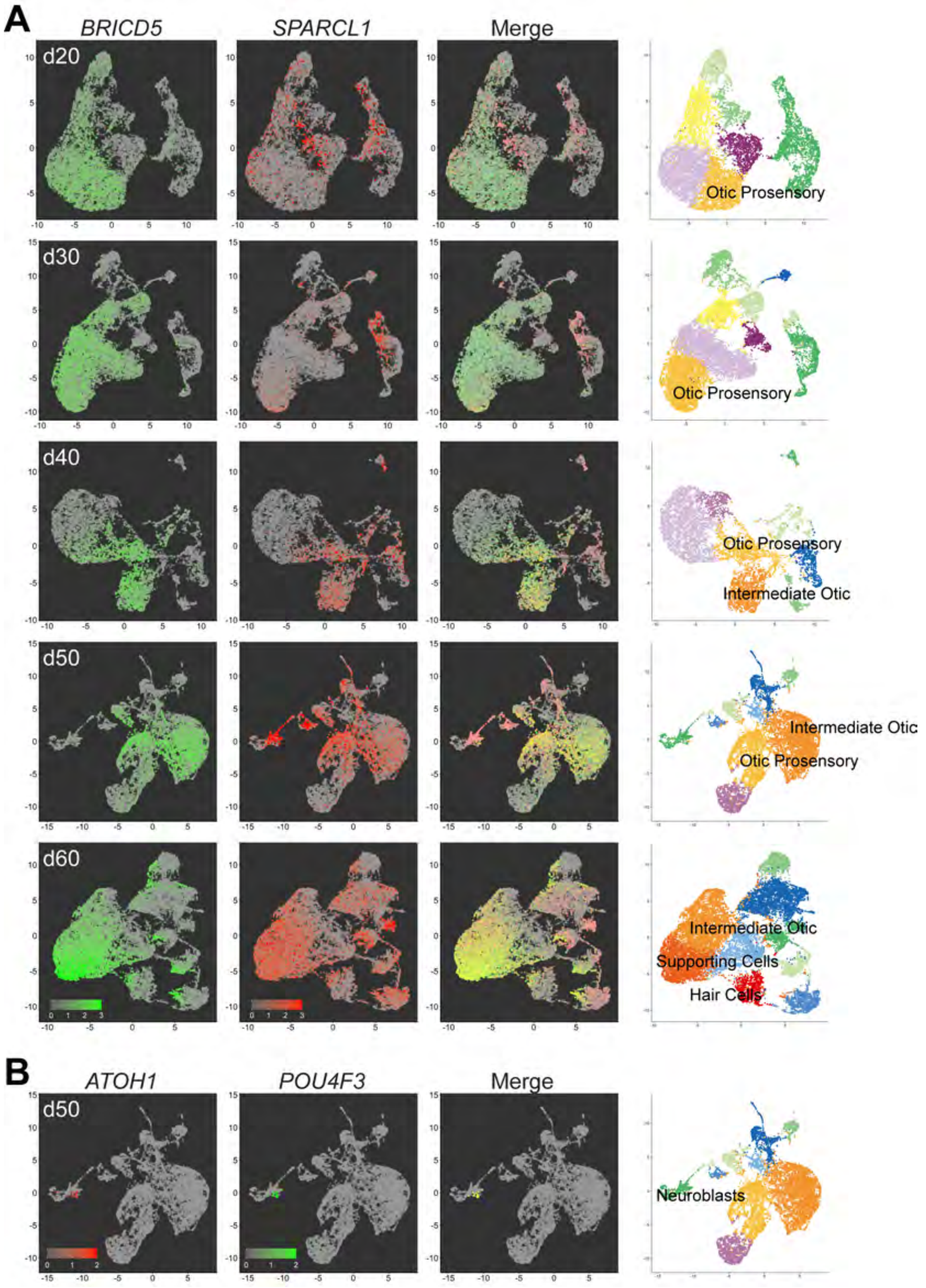

Fig. S4. Supporting cell and hair cell marker expression in human inner ear organoids.

(A) Expression of the supporting cell markers *BRICD5* and *SPARCL1* in individual datasets. (B) Identification of *ATOH1*$^{+}$ *POU4F3*$^{+}$ putative hair cells in the d50 dataset classified as a neuroblast cluster by unsupervised clustering.

Development • Supplementary information

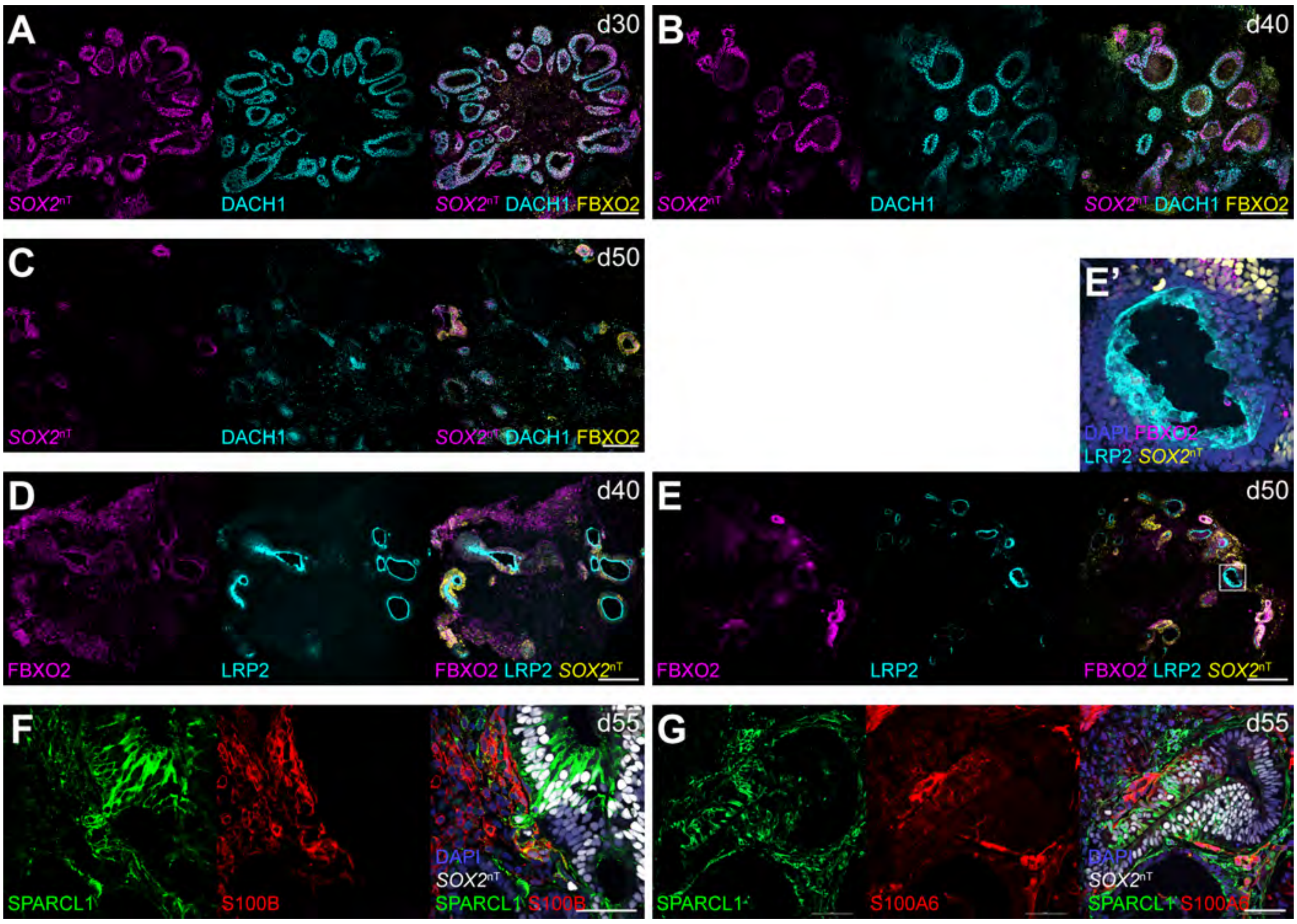

Fig. S5. Nonsensory otic cells in the human inner ear organoids

(A-C), DACH1 and FBXO2 expression at d30 (A), d40 (B), and d50 (C). (D-E) LRP2 and FBXO2 expression at d40 (D) and d50 (E; E' for the boxed region in E). (F-G) SPARCL1$^+$ S100B$^+$ (F) and SPARCL1$^+$ S100A6$^+$ surrounding the *SOX2*$^{ntdTomato+}$ vesicles. Scale bars: A-E, 200 μm; F-G, 50 μm.

Development • Supplementary information

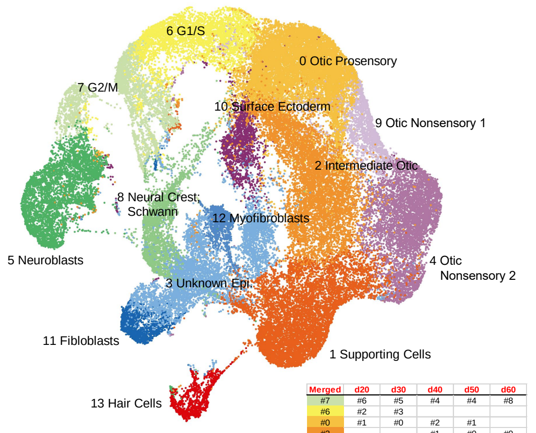

Merged	d20	d30	d40	d50	d60
#7	#6	#5	#4	#4	#8
#6	#2	#3			
#0	#1	#0	#2	#1	
#2			#1	#0	#0
#1					#2
#13					#5
#9	#0	#1	#5		
#4			#0	#2	
#10	#4	#6			
#5	#3 #5	#2	#7	#5	#7
#8	#7	#4	#6	#6	#6
#3				#7	#3
#11		#7	#3	#3	#1
#12				#8	#4

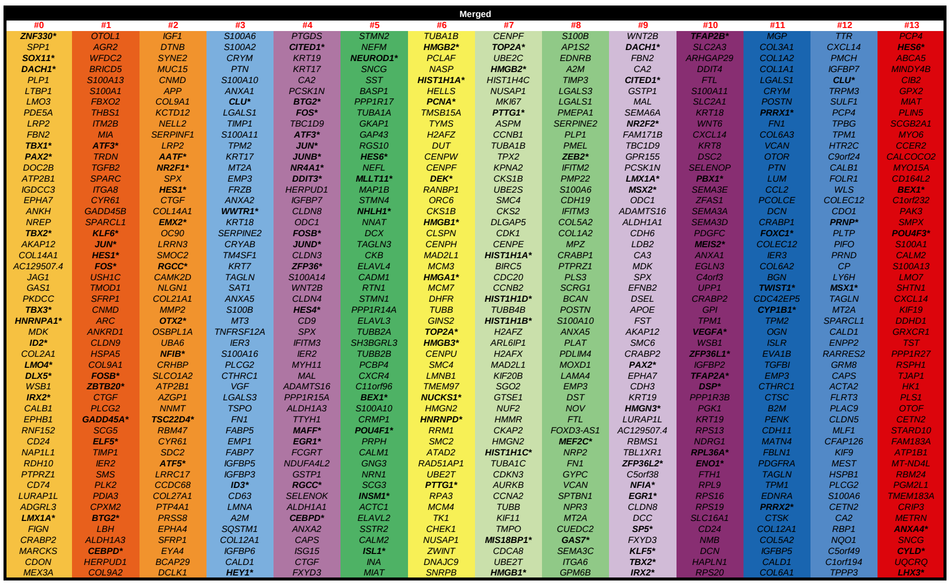

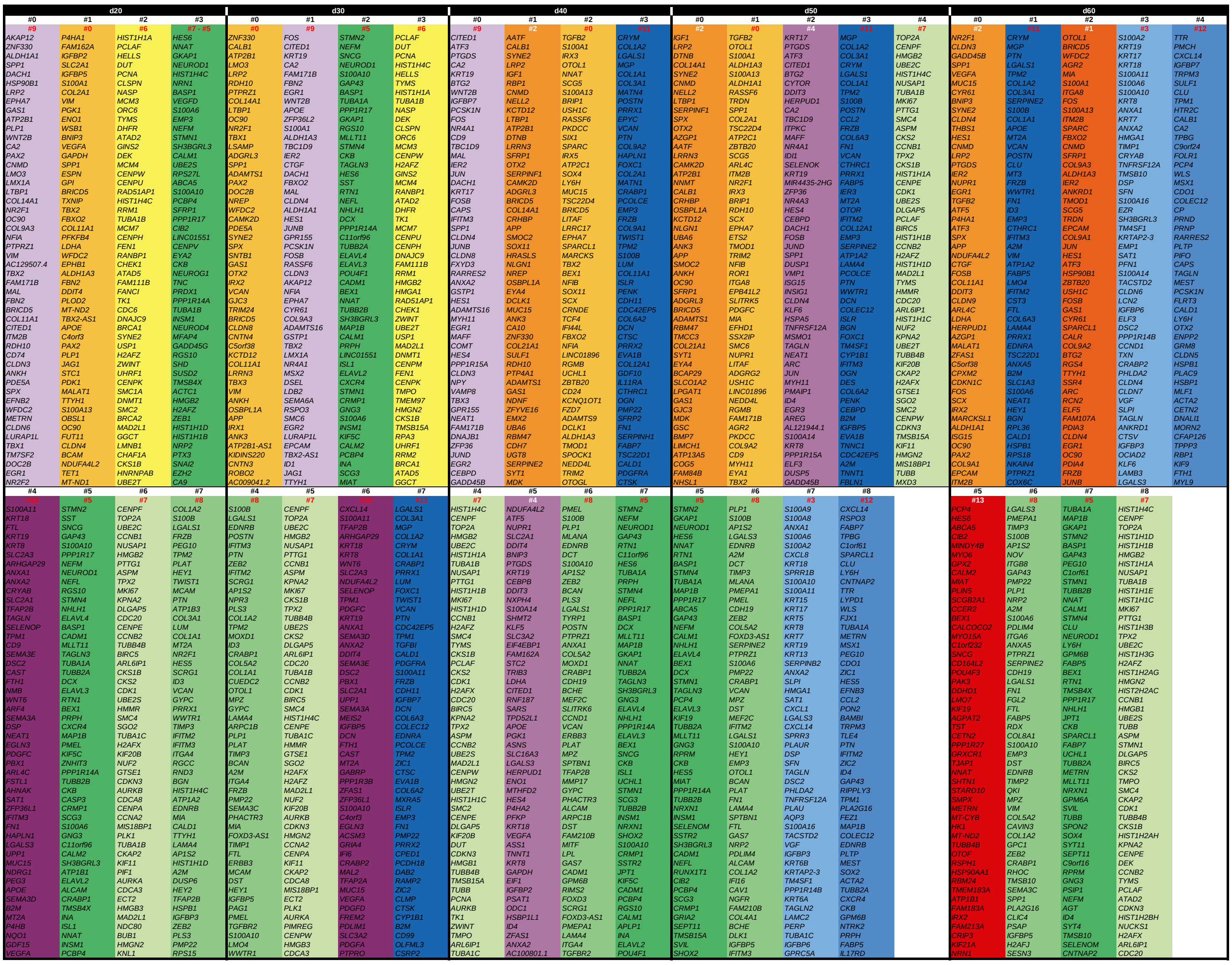

Fig. S6. List of differentially expressed genes (DEGs)

Each color on the UMAP plot (upper left) corresponds to each cluster in the merged dataset (upper right; transcription factors are highlighted with asterisks) and individual dataset (bottom).

Development • Supplementary information

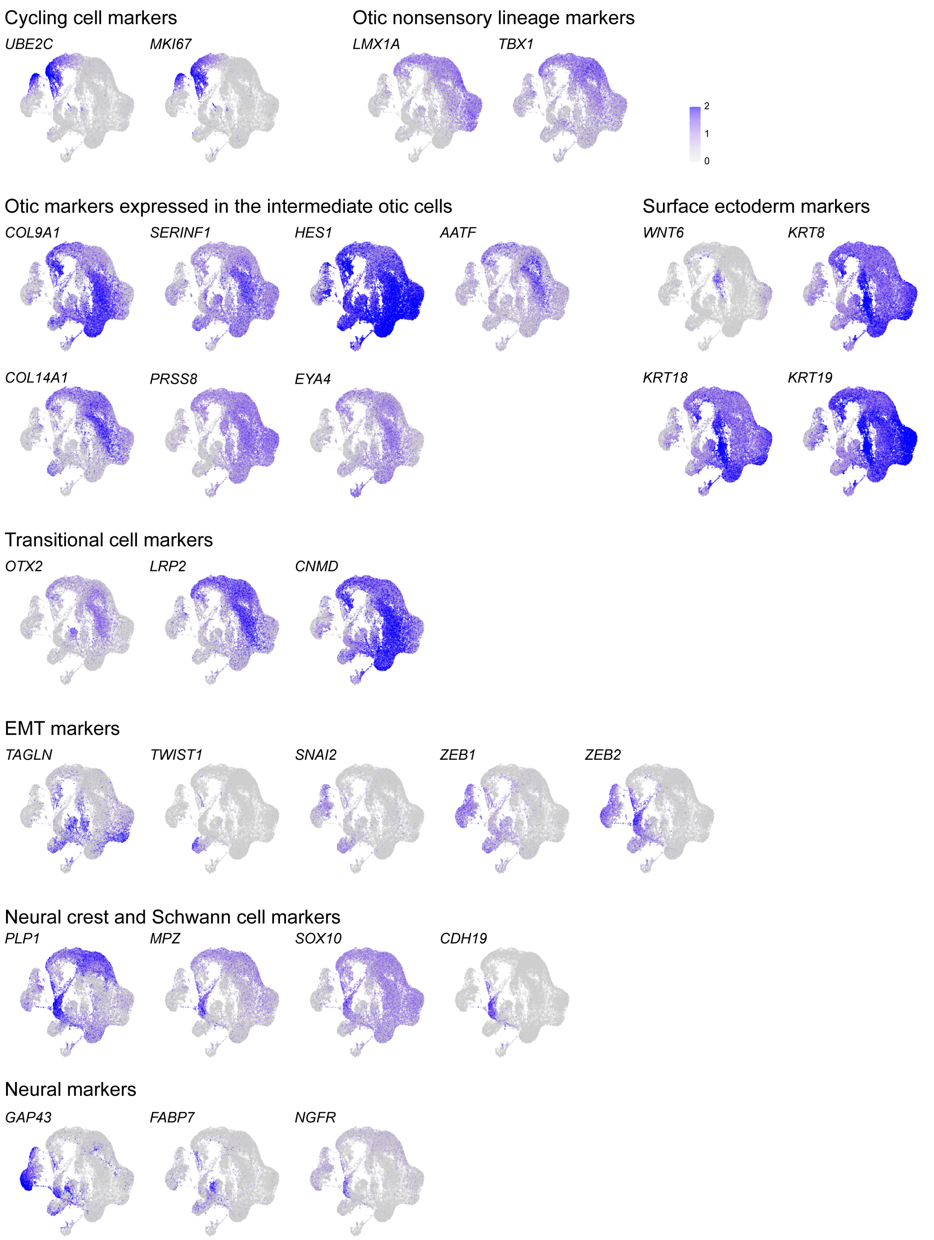

Fig. S7. Feature plots for otic lineage markers in the merged dataset.
Feature plots showing the expression of cycling markers (*UBE2C* and *MKI67*), otic nonsensory cell lineage markers (*LMX1A* and *TBX1*), otic markers expressed in the intermediate otic cells (*COL9A1*, *SERINF1*, *HES1*, *AATF*, *COL14A1*, *PRSS8*, *EYA4*), and transitional cell markers (*OTX2*, *LRP2*, and *CNMD*), surface ectodermal marker (*WNT6*) and cytokeratin markers (*KRT8*, *KRT18*, and *KRT19*), endothelial-to-mesenchymal transition markers (*TAGLN*, *TWIST1*, *SNAI2*, *ZEB1*, and *ZEB2*), neural crest and Schwann cell markers (*PLP1*, *MPZ*, *SOX10*, *CDH19*), and neural markers expressed in Schwann cells (*GAP43*, *FABP7*, *NGFR*).

Development • Supplementary information

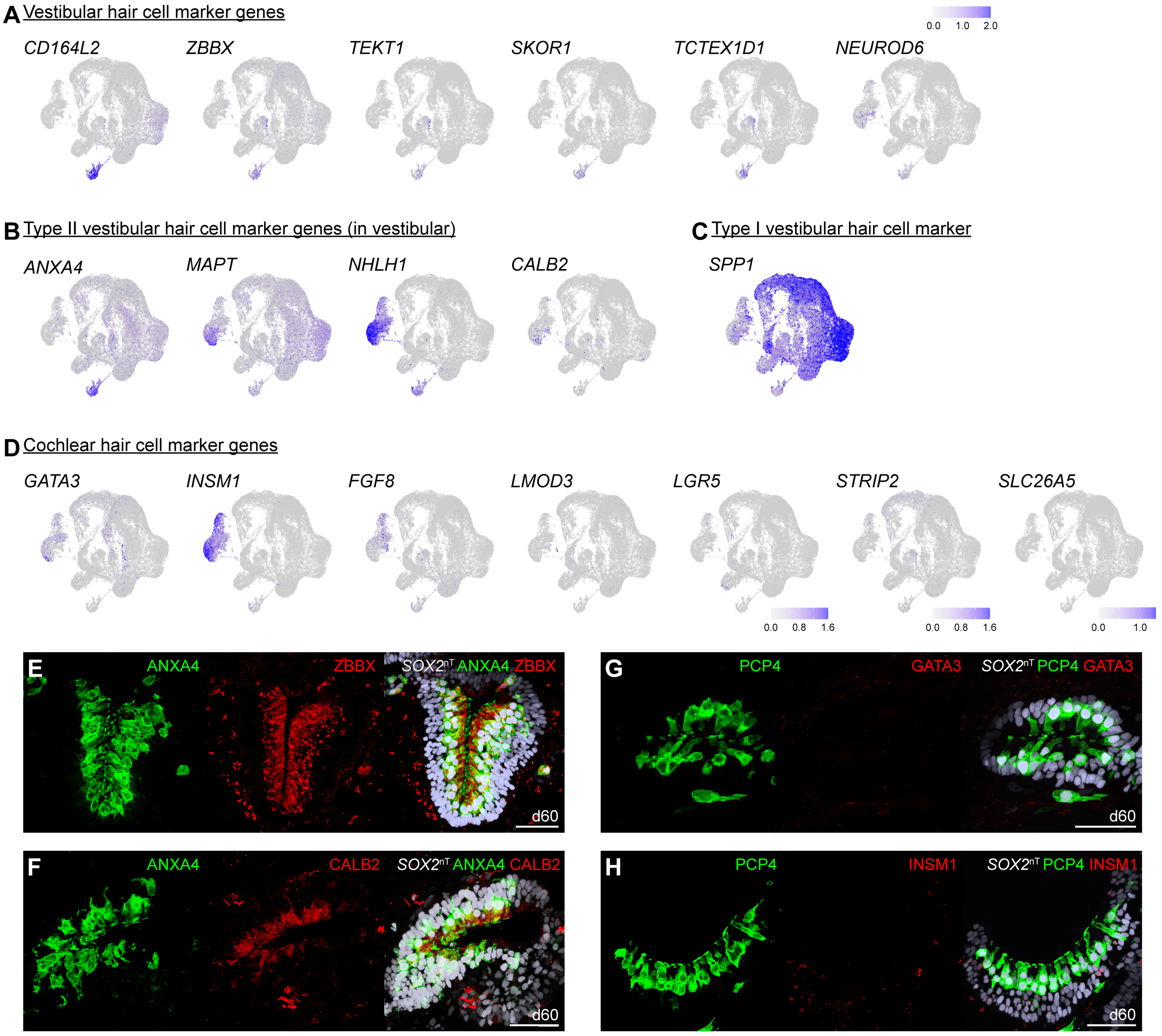

Fig. S8. Hair cell type identification in human inner ear organoids.

(A-D) Feature plots in the merged dataset for vestibular hair cell markers (A), type II vestibular hair cell markers (B), type I hair cell marker (C), and cochlear hair cell markers (D). (E-H) The presence of the type II hair cell markers ANXA4, ZBBX, and CALB2 (E,F) and the absence of the cochlear hair cell marker GATA3 and INSM1 (G,H). Scale bars: E-H, 50 µm

Development • Supplementary information

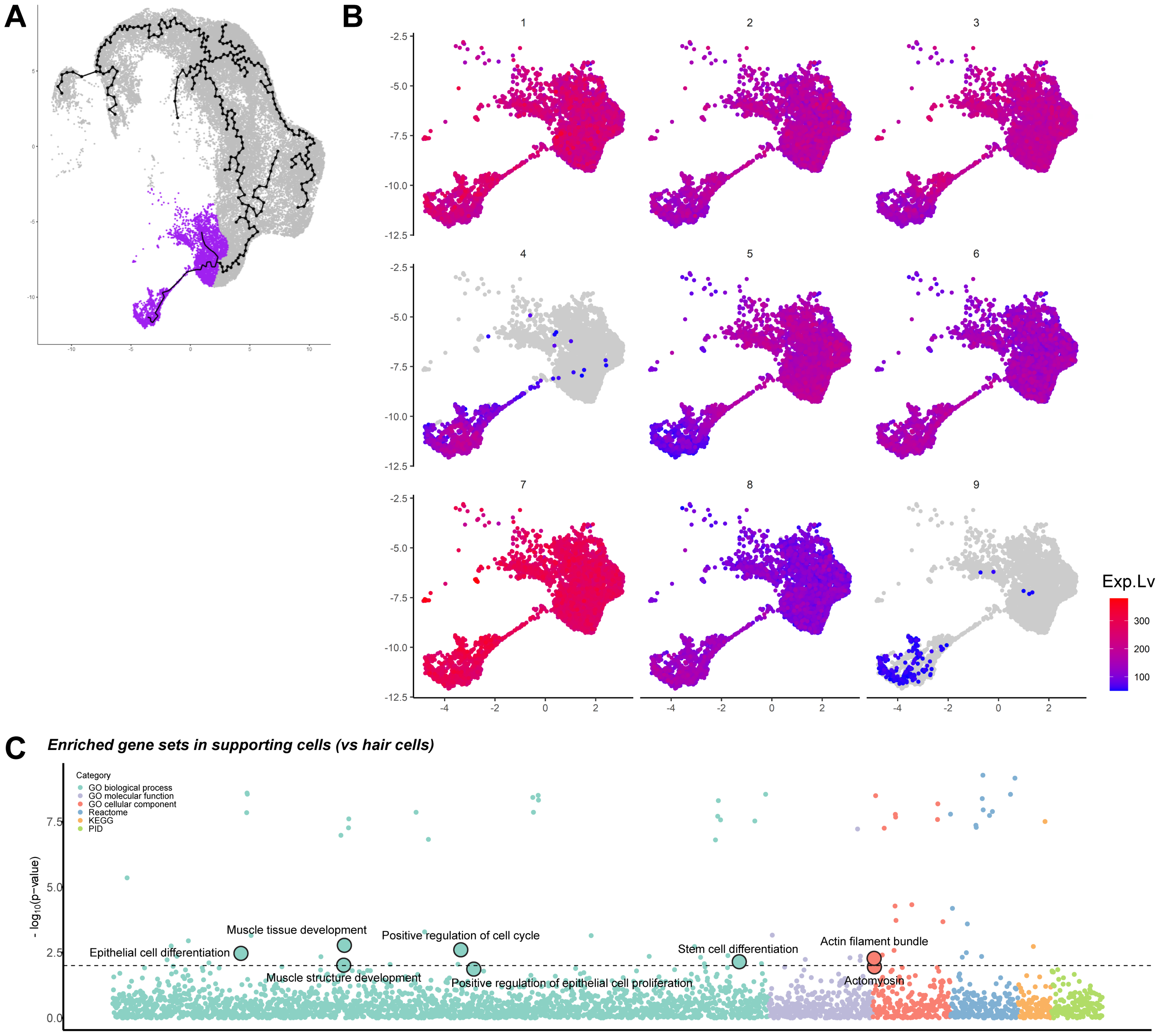

Fig. S9. Analysis of the supporting cell-hair cell trajectory.

(A) Selected cells in UMAP for identification of early upregulated genes in hair cells. (B) Gene expression patterns detected in the selected dataset in (A). (C) Bubble plot showing enriched gene sets in supporting cells in comparison to hair cells.

Development • Supplementary information

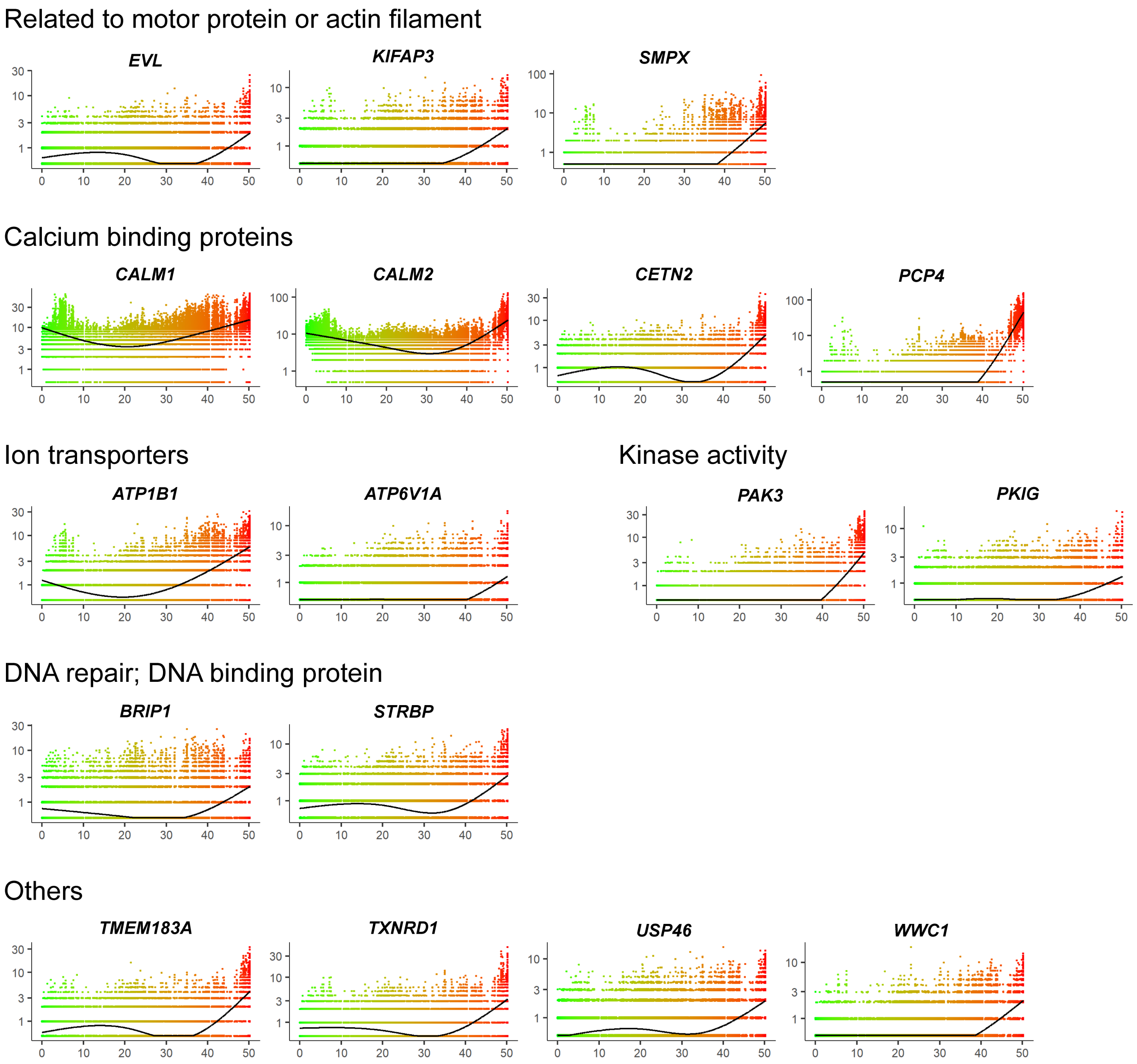

Fig. S10. Upregulated genes in hair cells vs. supporting cells (1).

Hair cell-enriched genes upregulated before the point 40 along the pseudotime. These genes are reported as hair cell-enriched genes in *Scheffer et al., 2015*.

Development • Supplementary information

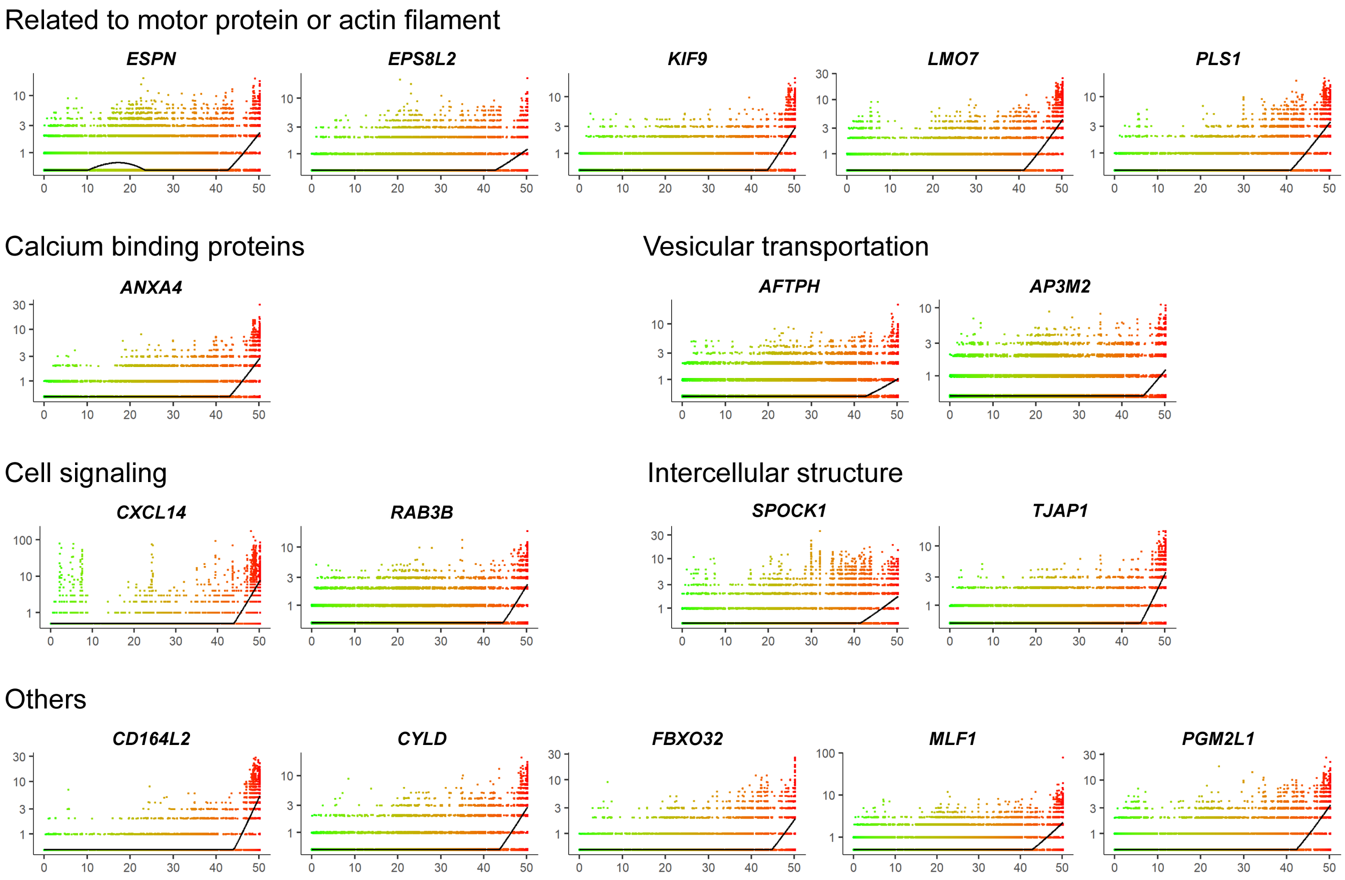

Fig. S11. Upregulated genes in hair cells vs. supporting cells (2).

Hair cell-enriched genes upregulated between the points 40 and 45 along the pseudotime. These genes are reported as hair cell-enriched genes in *Scheffer et al., 2015*.

Development • Supplementary information

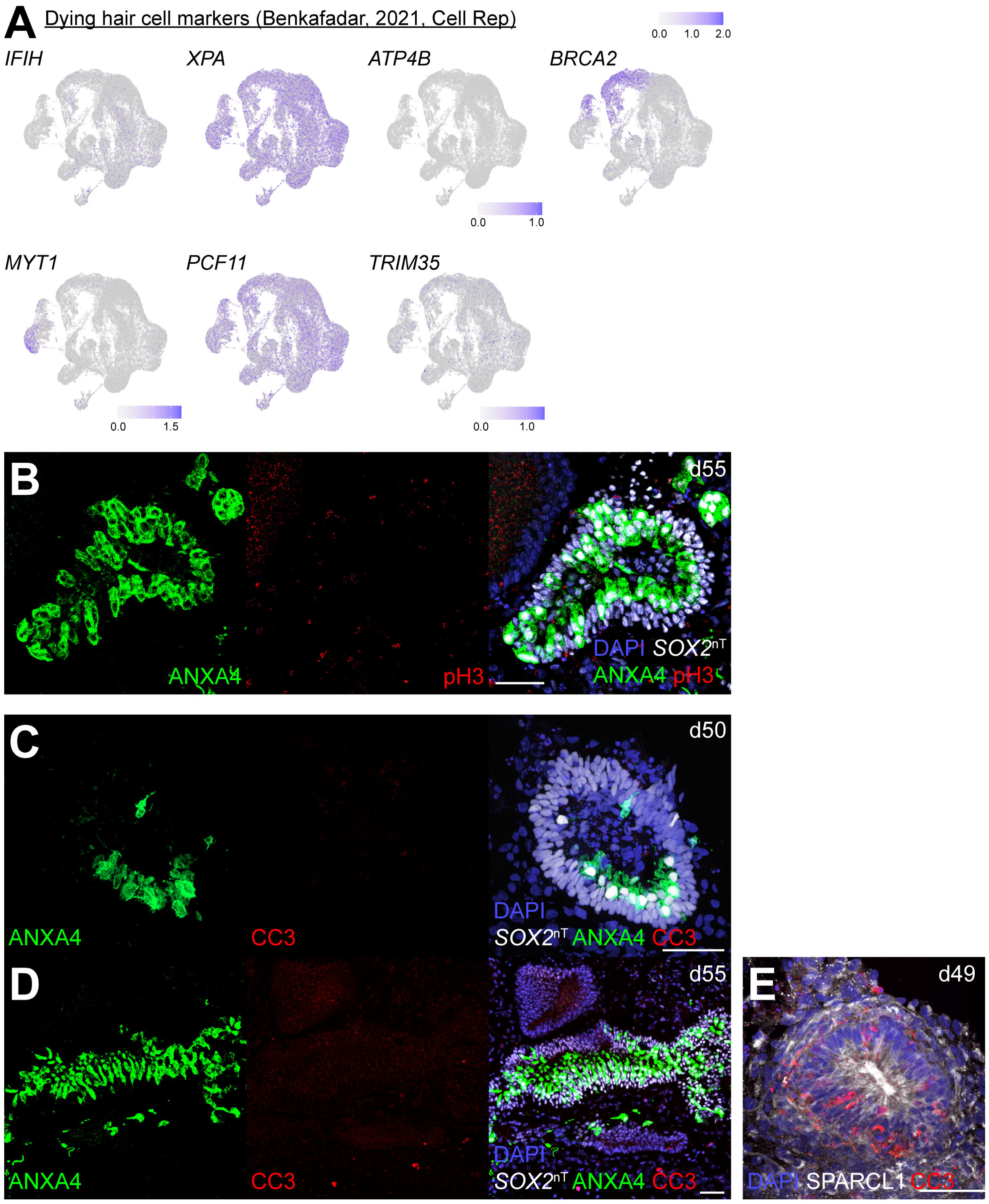

Fig. S12. Little sign of hair cell regeneration in human inner ear organoids before d60.
(A) Feature plots for dying hair cell marker genes (Benkafadar et al., 2021) in the merged dataset. (B-D) Immunofluorescence for the proliferation marker phosphorylated histone H3 (pH3) (B) and the apoptotic marker cleaved caspase 3 (CC3). (E) Clear CC3 expression is detected in human inner ear organoids undergoing drug-induced apoptotic cell death. Scale bars: B-E, 50 µm

Development • Supplementary information

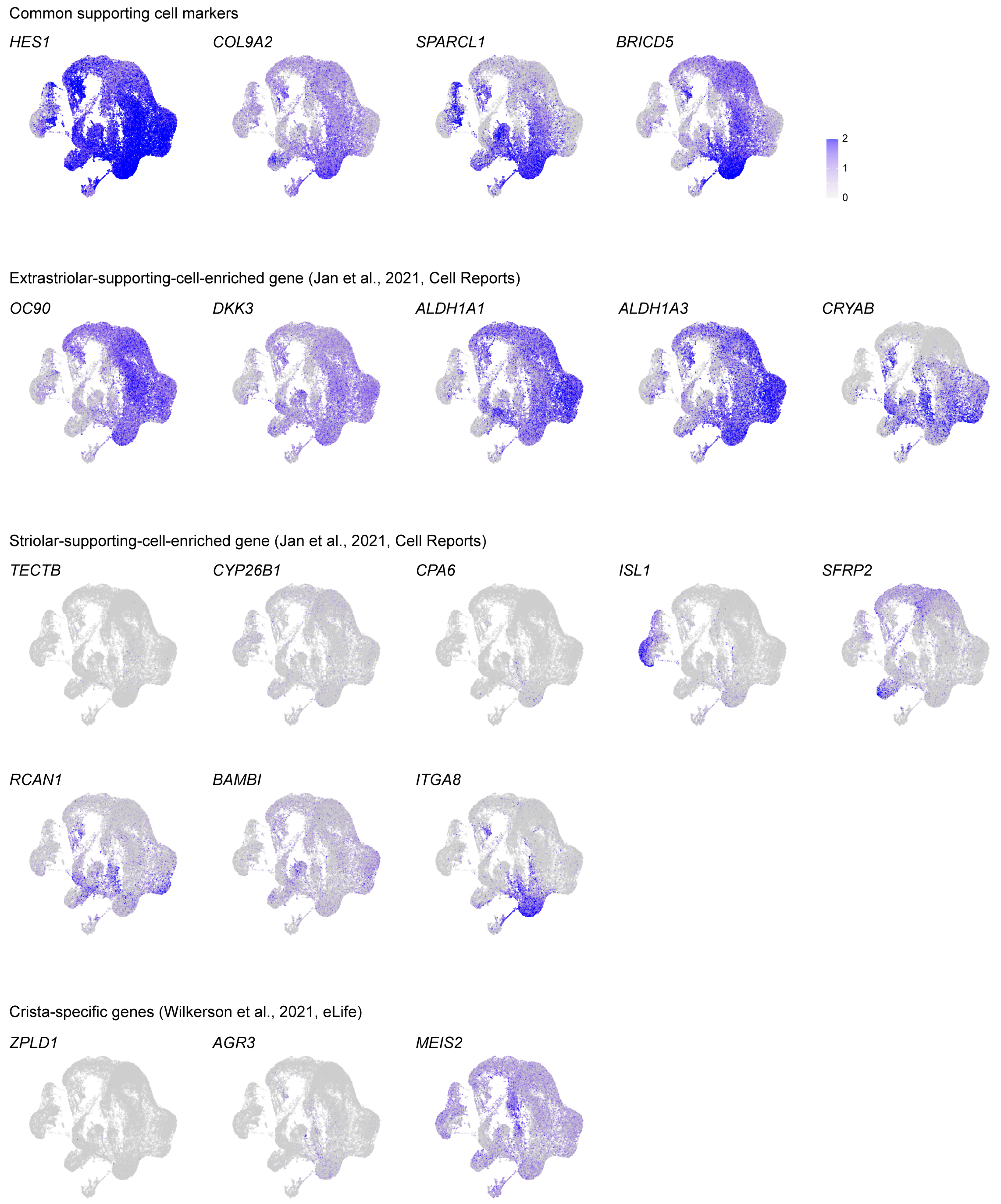

Fig. S13. Feature plots for supporting cell markers in the merged data set.

Feature plots showing the expression of supporting cell-expressing genes (*HES1*, *COL9A2*, and *SPARCL1*) as well as vestibular supporting cell marker (*BRICD5*), extrastriolar supporting cell-enriched genes (*OC90*, *DKK3*, *ALDH1A1*, *ALDH1A3*, and *CRYAB*), striolar supporting cell-enriched genes (*TECTB*, *CYP26B1*, *CPA6*, *ISL1*, *SFRP2*, *RCAN1*, *BAMBI*, and *ITGA8*), and crista specific genes (*ZPLD1*, *AGR3*, and *MEIS2*).

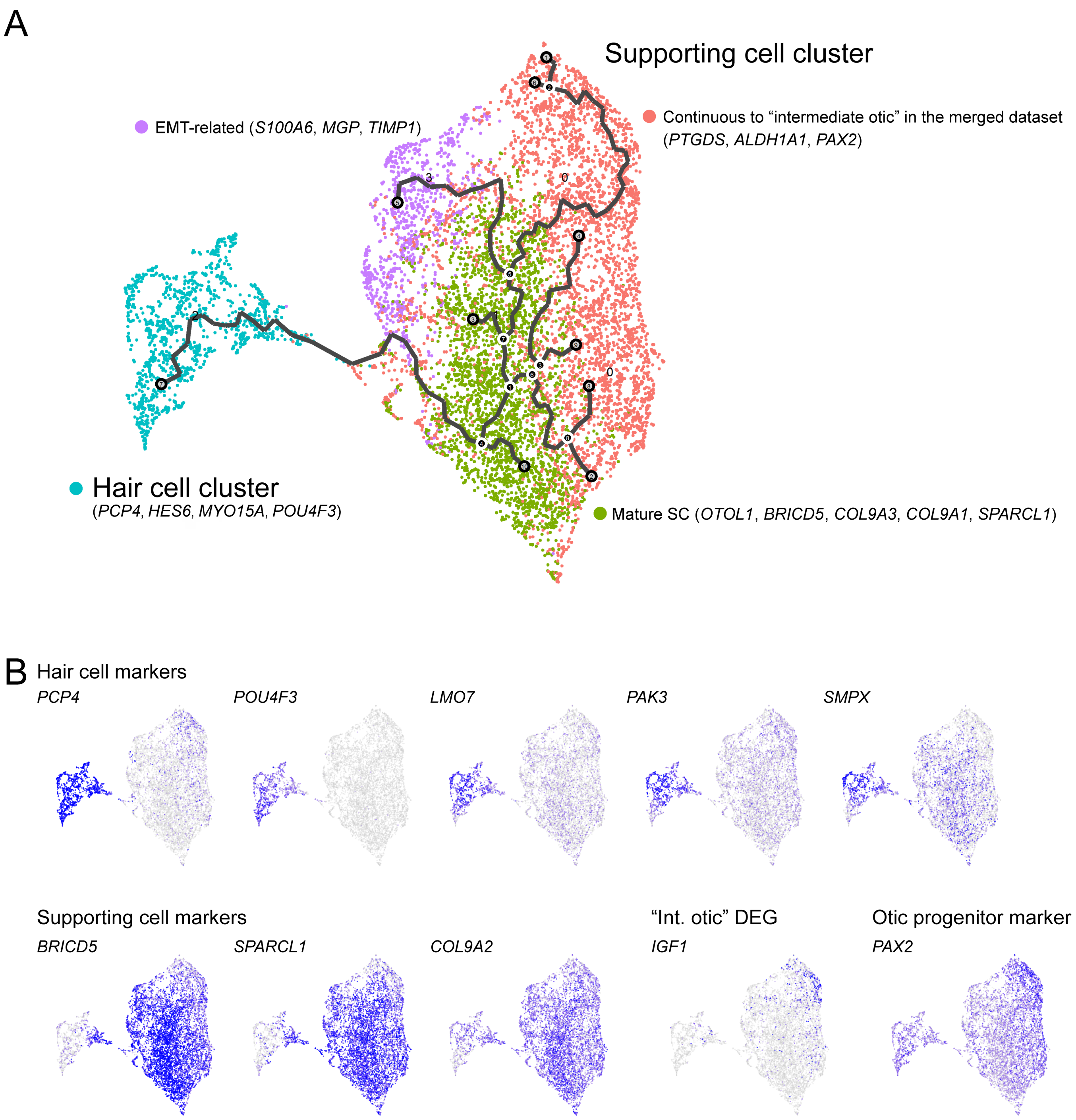

Fig. S14. Sub-clustering analysis for the supporting cell and hair cell clusters.

A UMAP plot showing sub-clusters and developmental trajectories in the supporting cell cluster. Also shown are feature plots for hair cell markers (*PCP4*, *POU4F3*, *LMO7*, *PAK3*, and *SMPX*), supporting cell markers (*BRICD5*, *SPARCL1*, and *COL9A2*), an enriched gene in the intermediate otic cells (*IGF1*), and otic progenitor marker (*PAX2*).

Development • Supplementary information

Table S1. List of primers used in Fig. 1.

Target	Amplicon Size		Sequence
Primer set 1	901 bp	Forward	5'-CAACCAGAAAAACAGCCCGGAC-3'
		Reverse	5'-CCTGTTGAAACAAGCAGGGGATGTC-3'
Primer set 2	1312 bp	Forward	5'-CACCAGGCACTGAAGCTGAAAGATG-3'
		Reverse	5'-CATGCAGGTTGACACCGTTGG-3'
Primer set 3	$SOX2^{nT}$, 3442 bp<br>WT, 1657 bp	Forward	5'-CAACCAGAAAAACAGCCCGGAC-3'
		Reverse	5'-CCATGCAGGTTGACACCGTTG-3'

Table S2. List of antibodies.

Target	Host	Isoform	Supplier	Cat#	Dilution
ANXA4	Goat	IgG	R&D Systems	AF4146	1:25
CALB2	Rabbit	IgG	Abcam	ab702	1:50
Cleaved Caspase 3	Rabbit	IgG	Cell Signaling	9664S	1:400
DACH1	Rabbit	IgG	Proteintech	10914-1-AP	1:100
FBXO2	Mouse	IgG1	Santa Cruz	sc-398111	1:25
GATA3	Goat	IgG	R&D Systems	AF2605	1:100
INSM1	Mouse	IgG1	Santa Cruz	sc-271408	1:100
LRP2	Rabbit	IgG	Abcam	ab76969	1:100
MYO6	Rabbit	IgG	Proteus	25-6791	1:100
MYO7A	Rabbit	IgG	Proteus	25-6790	1:100
PAX2	Mouse	IgG2a	Abnova	H00005076-M01	1:100
PAX8	Rabbit	IgG	Abcam	AB97477	1:100
PCP4	Rabbit	IgG	Invitrogen	PA5-52209	1:200
p-Histon 3	Rabbit	IgG	Cell Signaling	9701S	1:200
POU4F3	Mouse	IgG1	Santa Cruz	sc-81980	1:25
S100A6	Rabbit	IgG	Novus	NBP1-89388	1:1000
S100B	Rabbit	IgG	Abcam	NBP1-89388	1:100
SOX2	Mouse	IgG1	BD Biosciences	561469	1:100
SPARCL1	Goat	IgG	R&D Systems	AF2728	1:25
tdTomato	Goat	IgG	OriGene/SICGEN	AB8181-200	1:50
TUJ1	Mouse	IgG2a	BioLegend/Covance	801201	1:200
ZBBX	Rabbit	IgG	Invitrogen	PA5-57612	1:200
Oct-3/4 - AF488	(Rat)	(IgG2a)	eBioscience	53584182	1:100
SSEA4 - SyLight650	(Mouse)	(IgG3)	Invitrogen	MA1-021-D650	1:100
Mouse IgG2A - AF488	(Goat)	-	Invitrogen	A21131	1:1000
Rabbit IgG - AF488	(Donkey)	-	Invitrogen	A21206	1:1000
Goat IgG - AF488	(Donkey)	-	Invitrogen	A11055	1:1000
Goat IgG - AF546	(Goat)	-	Invitrogen	A11056	1:1000
Mouse IgG1 - AF647	(Goat)	-	Invitrogen	A21240	1:1000
Mouse IgG2A - AF647	(Goat)	-	Invitrogen	A21241	1:1000
Rabbit IgG - AF647	(Donkey)	-	Invitrogen	A31573	1:1000
Goat IgG - AF647	(Donkey)	-	Invitrogen	A21447	1:1000

Table S3. Lists of enriched gene sets in hair cells (vs. supporting cells) and supporting cells (vs. hair cells and vs. intermediate otic cells) used in Fig. 5 and Fig. S9.

Click here to download Table S3

Development • Supplementary information
