# Supplementary material for: Defining developmental trajectories of prosensory cells in human inner ear organoids at single-cell resolution
Source: Development. 2023 Jun 29;150(12):dev201071. doi: 10.1242/dev.201071 (PMC10323240; doi:10.1242/dev.201071)
Supplement: Supplementary file 1 [file develop-150-201071_review_history.pdf]

Defining developmental trajectories of prosensory cells in human inner ear organoids at single-cell resolution

Yoshitomo Ueda, Takashi Nakamura, Jing Nie, Alexander J Solivais, John R Hoffman, Becca J Daye and Eri Hashino
DOI: 10.1242/dev.201071

Editor: James Wells

Review timeline

Original submission:	26 July 2022
Editorial decision:	20 September 2022
First revision received:	22 February 2023
Editorial decision:	11 April 2023
Second revision received:	23 May 2023
Accepted:	24 May 2023

Original submission

First decision letter

MS ID#: DEVELOP/2022/201071

MS TITLE: Defining developmental trajectories of prosensory cells in human inner ear organoids at single-cell resolution

AUTHORS: Yoshitomo Ueda, Takashi Nakamura, Jing Nie, Alexander J Solivais, John R Hoffman, and Eri Hashino

I have now received all the referees' reports on the above manuscript, and have reached a decision. The referees' comments are appended below, or you can access them online: please go to BenchPress and click on the 'Manuscripts with Decisions' queue in the Author Area.

As you will see, the referees express considerable interest in your work, but have some significant criticisms and recommend a substantial revision of your manuscript before we can consider publication. If you are able to revise the manuscript along the lines suggested, which may involve further experiments, I will be happy receive a revised version of the manuscript. Your revised paper will be re-reviewed by one or more of the original referees, and acceptance of your manuscript will depend on your addressing satisfactorily the reviewers' major concerns. Please also note that Development will normally permit only one round of major revision. If it would be helpful, you are welcome to contact us to discuss your revision in greater detail. Please send us a point-by-point response indicating your plans for addressing the referee's comments, and we will look over this and provide further guidance.

Please attend to all of the reviewers' comments and ensure that you clearly highlight all changes made in the revised manuscript. Please avoid using 'Tracked changes' in Word files as these are lost in PDF conversion. I should be grateful if you would also provide a point-by-point response detailing how you have dealt with the points raised by the reviewers in the 'Response to Reviewers' box. If you do not agree with any of their criticisms or suggestions please explain clearly why this is so.

© 2023. Published by The Company of Biologists under the terms of the Creative Commons Attribution License (https://creativecommons.org/licenses/by/4.0/).

Reviewer 1

Advance summary and potential significance to field

This is an interesting manuscript that examines the trajectories of Sox2-expressing otic cells in organoids derived from human ES cells. This group has previously established that they can generate otic organoids from human ES cells that produce type II vestibular hair cells and supporting cells by day 60. For this study, the authors generate a Sox2 reporter line and perform single-cell RNAseq at 10 day intervals from d20-60. The novel finding from this data is that pseudotime analysis indicates that the hair cells arise from a population of supporting cells, rather than bipotential progenitor cells, as has been suggested from the literature. Overall, the study is well performed with in-depth analyses of each time point and cell population. The weakness of the study is its reliance on scRNAseq characterization of cell populations to generate conclusions. However, this is a unique study of human otic cell populations that has generated novel and potentially important results.

Comments for the author

Specific comments

It does not appear, from Fig 2A, that either supporting cells or hair cells are present at d50, and both populations appear at d60. Moreover, there is a large otic prosensory population at d50 that disappears by d60. In addition, there is an unknown epithelium at P60 (light blue), that shows some overlap with the hair cell population. These data suggest some dramatic changes are taking place between d50 and d60, that are particularly relevant to the conclusions of this study. Thus, it would be revealing to perform an analysis at d55, to gain better resolution as to the changes taking place in these populations.

In the discussion pg 16, (line 10-11), the authors indicate there are supporting cells at d50, although the data in Fig 2A do not show supporting cells at this time.

The data suggests that changes in Wnt signaling may be a unique feature in the production of hair cells. It would be interesting to test this (for example at d50 using an inhibitor) to provide further support for whether Wnt signaling is a critical factor in hair cell generation.

The study suggests that hair cells may arise from supporting cells, rather than from bipotential progenitors that give rise to both hair cells and supporting cells. It is well known that hair cells can arise from supporting cells, for example during regeneration. Thus, it is unclear whether the data here reflect normal development or, given the culture condition, whether the hair cells are arising through a regenerative process. Based on the expression analysis, are there are studies that may support the idea of a developmental process rather than a regenerative one? In any case this possibility should be discussed.

One of the drawbacks to this study is whether the in vitro system reflects the in vivo process well, and therefore gives insight into human otic development. In the discussion the authors cite a few papers that suggest in vivo support for the idea that hair cells arise from supporting cells rather than bipotential precursors, although it is not clear what this support is, or how strongly it supports the in vitro data.

The supporting cell analysis indicates that these are likely extrastriolar supporting cells. These cells are known to give rise to new hair cells quite late, for example in the mouse these give rise to the hair cells arising postnatally. Thus, is it possible this is a distinct lineage that may not reflect general hair cell and supporting cell development?

The authors use the term intermediate otic cells, to suggest a population that lies between the otic prosensory population and the supporting cell population.

© 2023. Published by The Company of Biologists under the terms of the Creative Commons Attribution License (https://creativecommons.org/licenses/by/4.0/).

However, this term is not used in the images form the UMAP plots, and is instead defined generically as otic epithelial cells (Fig 2 and 3). It would be helpful to define and label this population more specifically in the figures.

Reviewer 2

Advance summary and potential significance to field

The manuscript by Ueda et al. reports the developmental trajectories of the prosensory cells in human inner ear organoids by scRNA-seq analyses. The authors have pioneered inner ear organoid research, including from both mouse and human ES cells. This study described an interesting hypothesis that hair cells of human origin may be derived by some of the supporting cells rather than a bi-fated progenitor cells. The study also suggests the presence of intermediate otic-lineage cells that give rise to the supporting cells; and also highlights specific regulation of ion channel activities and wnt signaling pathways in supporting cells and hair cells, respectively. Overall, the manuscript provides a first piece of clue to how human hair cells develop in an organoid model, which may have implication in human hair cell development and regeneration in vivo.

Comments for the author

The manuscript by Ueda et al. reports the developmental trajectories of the prosensory cells in human inner ear organoids by scRNA-seq analyses. The authors have pioneered inner ear organoid research, including from both mouse and human ES cells. This study described an interesting hypothesis that hair cells of human origin may be derived by some of the supporting cells rather than a bi-fated progenitor cells. The study also suggests the presence of intermediate otic-lineage cells that give rise to the supporting cells; and also highlights specific regulation of ion channel activities and wnt signaling pathways in supporting cells and hair cells, respectively. Overall, the manuscript provides a first piece of clue to how human hair cells develop in an organoid model, which may have implication in human hair cell development and regeneration in vivo.

However, the bulk of the study is based on scRNA-seq analyses, some of the claims should be substantiated and clarified by additional analyses and immunostaining validations.

Major comments:
1. Probably the most significant novelties of this study is to identify the intermediate oti-lineage that give rise to supporting cells, which then differentiate into hair cells. This is in contrast with the default idea that bi-fated progenitors can either differentiate into supporting cells or hair cells. However, if the supporting cells differeniate directly to hair cells, there should be a transitional population that express both well-established supporting cell markers and immature (or mature) hair cell markers. This may be true with careful analyses of the scRNA-seq data, but validation of these transitional population using immuno-fluorescent co-staining of these markers will be necessary.
2. The scRNA-seq was performed with FACS-ed Sox2+ cells, which is understandable but may miss out potential Sox2- sensory cells. The authors should discuss this in the text. In addition, the authors should specify in the Abstract that the hair cells are “vestibular type II-like hair cells”, because it is unknown if vestibular type I or cochlear hair cells develop with the same trajectory.
3. The finding that some of the hair cell markers (Myo6, Pcp4 etc) were expressed earlier than pioneer transcription factors such as Atoh1 and Pou4f3 is interesting, surprising and maybe thought provoking. Again, this should be experimentally validated by immuno-staining analyses of the p60 organoids to check if some of the hair cells express Myo6 etc but not Atoh1 or Pou4f3.
4. The presence of putative hair cells in d50 organoids is also interesting, how are they different from d60 in term of transcriptome? Are these d50 hair cells simply fate determined early or represent a distinct subtype of hair cells? A comparative analysis of d50 and d60 hair cells will be highly informative.

Minor comments:
1. Expression of Sox2 should also be included in the feature plots, to show if various cell types show differences in Sox2 expression.

© 2023. Published by The Company of Biologists under the terms of the Creative Commons Attribution License (https://creativecommons.org/licenses/by/4.0/).

2. Fig. S4 and S5, NGFR and GAP43 are neuronal markers, which may show some expression in other cell types such as Schwann cells, however, they should be labelled as “neural markers” rather than “Schwann cell markers” to avoid confusion.
3. Page 8, line 2, “maintained until d40”, however, Fig. 1J shows d35.

Reviewer 3

Advance summary and potential significance to field

Ueda et al. provide much-needed insight into the cellular lineages emerging in human inner ear organoid cultures. Inner ear (or otic) organoid cultures have high complexity relative to other organoid systems, so the application of single-cell approaches is needed to illuminate the diverse cell populations. Moreover, our knowledge of cell lineage decisions among otic epithelial cells—the key targets for inner ear therapies—is lacking. The authors aim to use these data to provide actionable insights for regenerative inner ear therapeutics. Here, the author’s narrowed their analysis to SOX2+ cell populations from various time points between days 30-80, which limited the dataset to the sensory portions of the otic lineage (supporting cells and hair cells), neural crest cells, and some CNS-like cells.

Overall, the analysis is thorough and well presented. The resulting data clarify the emergence of otic lineage cells in these cultures and largely reconfirm previous findings regarding the vestibular identity of organoids grown in this platform. A key weakness would be the lack of truly novel insights into the otic organoid culture cellular composition or the mechanisms of mammalian inner development, in general. Nonetheless, the dataset represents a valuable resource for anyone wishing to use otic organoids for their research.

Comments for the author

General:

As stated above, the analyses are thorough and executed following current bioinformatic standards. The visualizations are particularly well organized for readability.

This study aimed to identify previously unrecognized genes or gene sets that are upregulated in early-stage human hair cell differentiation. The expression of ion channel and ion transporter-related proteins as well as the involvement of the WNT pathway is not surprising as this has been described during development. In revising the manuscript, the authors should highlight potentially novel insights.

Do the data implicate other signaling pathways that have received less attention than the WNT pathway? An overview analysis of signaling pathway mediators expressed in the otic epithelial cells would be a good community resource.

Moreover, the expressed goal of the study seemed to be to identify new transcription factors that may control otic development, yet these insights are lacking. A curated list of differentially expressed TFs could also be an excellent resource for the community.

The authors use recent data on mouse inner ear development for comparison to human inner ear organoids, yet there is no discussion of the possible developmental differences between the mouse and human inner ear (on a scRNAseq level). In line with this, there is no validation of the RNAseq data: as in the confirmation of RNA/protein expression by IHC or ISH. Although a complete validation is unnecessary, some validation, particularly of novel markers would strengthen the manuscript.

Concerns about accessibility: It’s become typical with these types of studies to provide easy access to the data for readers less skilled in data science, either through a custom (CellxGene) or central (gEAR) web portal. The authors should use these resources to improve the impact of the study.

© 2023. Published by The Company of Biologists under the terms of the Creative Commons Attribution License (https://creativecommons.org/licenses/by/4.0/).

Moreover, spreadsheets containing the complete differential expression analysis, marker gene, and gene ontology data should accompany the paper.

Finally, the manuscript would benefit from a more targeted discussion of the weaknesses of the experimental design to aid the reader in interpreting the results in comparison to similar studies. For instance, the lack of biological replicates or alternate cell lines should be discussed. Was the depth of sequencing sufficient to address the author's questions? These factors do not devalue the work, but rather need to be stated clearly.

Minor:
6.12 The presence of type II vestibular hair cells is not shown in this manuscript, and there seems to be no effort to evaluate the types of hair cells using the RNA-seq data or histology. The authors should discuss this omission or include a deeper analysis of hair cell type.

7.2 The name of the hESC cell line used is not provided clearly.

Fig.1 L-L'': The SOX2 expression in the putative supporting cells is overexposed.

Fig.2 A: Please include the number of cells captured per timepoint.

Fig.2 C: The putative endolymphatic duct population and immature dark cell population show very similar gene expression at d40. Are these cell types distinct? Furthermore, at d50-60 these populations are not shown. Please discuss.

Fig.3 E: See above.

15.6 Vestibular identity of HCs is not shown in this manuscript.

17.3-5 Reword sentence for clarity.

19.5 Transitional epithelial cells and dark cells are distinct cell types.
Merging of datasets: has an algorithm for the integration of datasets been used?
For instance, Harmony or CCA? Were multiple methods tested to assess over-correction?

26.16 Missing symbols

First revision

Author response to reviewers' comments

Dear Reviewers:

We appreciate your interest in our work and prompt and comprehensive reviews of our manuscript. In response to your suggestions, we have revised the manuscript, by reorganizing the main and supplementary figures as outlined below, and adding one main figure, four additional supplementary figures and a supplementary table. We believe the changes we made have strengthened the quality and clarity of our study. We apologize that we needed a long time for the revision due to the necessity of obtaining new data from day 30 – 60 cultures for immunofluorescence and small molecule administration. We very much appreciate your review of this revised manuscript.

Reviewer 1
Advance Summary and Potential Significance to Field: *This is an interesting manuscript that examines the trajectories of Sox2-expressing otic cells in organoids derived from human ES cells. This group has previously established that they can generate otic organoids from human ES cells*

© 2023. Published by The Company of Biologists under the terms of the Creative Commons Attribution License (https://creativecommons.org/licenses/by/4.0/).

that produce type II vestibular hair cells and supporting cells by day 60. For this study, the authors generate a Sox2 reporter line and perform single-cell RNAseq at 10 day intervals from d20-60. The novel finding from this data is that pseudotime analysis indicates that the hair cells arise from a population of supporting cells, rather than bipotential progenitor cells, as has been suggested from the literature. Overall, the study is well performed with in-depth analyses of each time point and cell population. The weakness of the study is its reliance on scRNAseq characterization of cell populations to generate conclusions. However, this is a unique study of human otic cell populations that has generated novel and potentially important results.

Response: Thank you very much for your endorsement and valuable feedback on our manuscript.

Specific comments:
It does not appear, from Fig 2A, that either supporting cells or hair cells are present at d50, and both populations appear at d60. Moreover, there is a large otic prosensory population at d50 that disappears by d60. In addition, there is an unknown epithelium at P60 (light blue), that shows some overlap with the hair cell population. These data suggest some dramatic changes are taking place between d50 and d60, that are particularly relevant to the conclusions of this study. Thus, it would be revealing to perform an analysis at d55, to gain better resolution as to the changes taking place in these populations.

Response: Both putative hair cells and supporting cells exist at d50, although they are not segregated as distinct clusters (Fig. S4) perhaps due to a small number of hair cells and relatively weak marker expression in supporting cells. We speculate that the disappearance of the otic prosensory population in the d60 dataset is because of the selection of strong *SOX2*^{ntdTomato} positive cells to increase the hair cell number as we stated in the Materials and Methods section. Our data from *PAX2*^{nGFP}:*POU4F3*^{ntdTomato} organoids show weak *PAX2*$^{nGFP+}$ expressing vesicles at d60, strongly suggesting the existence of otic progenitors in d60 organoids (data not shown). As for the unknown epithelium, we found that SPARCL1^{+} S100B^{+} or SPARCL1^{+} S100A6^{+} cells in the transition to the basal area of epithelia at d55 (Fig. S5F,G) suggesting the conversion to different types of supporting cells or the epithelium to mesenchymal transition (EMT) although detailed investigation awaits further characterization. We have included these findings and limitations to our current study in the result (P10, L1-6) and discussion (P21, L9 – P23, L2) sections.

In the discussion pg 16, (line 10-11), the authors indicate there are supporting cells at d50, although the data in Fig 2A do not show supporting cells at this time.

Response: To reveal the existence of supporting cell populations in the individual datasets, we have included feature plots demonstrating co-expression of *SPARCL1* and *BRICD5* (Fig. S4A). Despite the existence of *SPARCL1*$^{+}$/*BRICD5*$^{+}$ cells in the d40-60 datasets, the cell clustering algorithm with a resolution recommended by the Seurat pipeline failed to identify obvious supporting cell clusters in the d40 and d50 datasets presumably due to similar gene expression with other otic lineage clusters (Fig. S3A and Fig. S6).

The data suggests that changes in Wnt signaling may be a unique feature in the production of hair cells. It would be interesting to test this (for example at d50 using an inhibitor) to provide further support for whether Wnt signaling is a critical factor in hair cell generation.

Response: Based on the reviewer's suggestion, we grew organoids in the presence or absence of the potent Wnt inhibitor IWP2 starting on d30, since *POU4F3*$^{ntdTomato+}$ putative hair cells arise as early as d35 with our current protocol (Moore et al., 2022). The IWP2 treatment significantly decreased the number of *POU4F3*$^{ntdTomato+}$cells (Fig. 6). This strongly suggests that Wnt signaling is critical for hair cell generation in human inner ear organoids. We have included these new results (P16, L1-9).

The study suggests that hair cells may arise from supporting cells, rather than from bipotential progenitors that give rise to both hair cells and supporting cells. It is well known that hair cells can arise from supporting cells, for example during regeneration. Thus, it is unclear whether the

© 2023. Published by The Company of Biologists under the terms of the Creative Commons Attribution License (https://creativecommons.org/licenses/by/4.0/).

data here reflect normal development or, given the culture condition, whether the hair cells are arising through a regenerative process. Based on the expression analysis, are there are studies that may support the idea of a developmental process rather than a regenerative one? In any case this possibility should be discussed.

Response: We assessed whether hair cells in inner ear organoids express some of dying hair cell markers identified in a recent study (Benkafadar et al., 2021). Hair cells in our dataset do not show distinct expression of the dying hair cell markers *IFIH*, *XPA*, *ATP4B*, *BRCA2*, *MYT1*, *PCF11* or *TRIM35* (Fig. S14A). Moreover, hair cell regeneration is triggered by apoptotic hair cell death, followed by proliferation of supporting cells (Wang et al., 2015, Benkafadar et al., 2021, Shu et al., 2019, Bramhall et al., 2014); however, we did not detect the expression of cleaved-caspase3 in hair cells as well as the proliferation marker phosphorylated-histone3 (pH3) in hair cells or supporting cells at d50 and d55 (Fig. S14B-D). Thus, it is unlikely that hair cells in our samples arise as a result of regeneration. We have included this intepretension in the discussion section (P17, L12 – P18, L2).

One of the drawbacks to this study is whether the in vitro system reflects the in vivo process well, and therefore gives insight into human otic development. In the discussion the authors cite a few papers that suggest in vivo support for the idea that hair cells arise from supporting cells rather than bipotential precursors, although it is not clear what this support is, or how strongly it supports the in vitro data.

Response: A recent *in vivo* scRNA-seq study (Jan et al., 2021) suggested that hair cells are differentiated from intermediate supporting cells in chicks, and another study stated that hair cells arise from supporting cell-like progenitors in mice (Burns et al., 2015). These previous studies are consistent with our current data and support the idea that hair cells could arise from intermediate supporting cell populations in humans during development, although additional experiments, such as lineage-tracing, will be necessary to verify this hypothesis. We have rephrased this part in the discussion section (P18, L6-9).

The supporting cell analysis indicates that these are likely extrastriolar supporting cells. These cells are known to give rise to new hair cells quite late, for example in the mouse these give rise to the hair cells arising postnatally. Thus, is it possible this is a distinct lineage that may not reflect general hair cell and supporting cell development?

Response: Based on our analysis, hair cells in d50-60 human inner ear organoids are developmentally equivalent to hair cells in humans at gestational weeks 8-12 (Moore et al., 2022), and this period is equivalent to E13.5-18 (Roccio and Edge, 2019) in mice, which is before postnatal hair cell trans-differentiation (Wang et al., 2015). Thus, the differentiation from supporting cells to hair cells seen in the human inner ear organoid is potentially a part of normal otic development although it is insufficient to completely exclude the presence of common otic progenitors. The question of whether dual-fated common progenitors exist in our system and how progenitors and supporting cells contribute to hair cell differentiation awaits future studies using more mature organoid samples.

The authors use the term intermediate otic cells, to suggest a population that lies between the otic prosensory population and the supporting cell population. However, this term is not used in the images form the UMAP plots, and is instead defined generically as otic epithelial cells (Fig 2 and 3). It would be helpful to define and label this population more specifically in the figures.

Response: We have changed the labeling "Otic Epithelium" to "Intermediate Otic (Cells)" in the UMAP plots and the dot plots in Fig. 2 and Fig. 3. We have also changed the description in the text for consistency.

Reviewer 2

Advance Summary and Potential Significance to Field and Comments for the Author:

The manuscript by Ueda et al. reports the developmental trajectories of the prosensory cells in human inner ear organoids by scRNA-seq analyses. The authors have pioneered inner ear organoid research, including from both mouse and human ES cells. This study described an interesting

© 2023. Published by The Company of Biologists under the terms of the Creative Commons Attribution License (https://creativecommons.org/licenses/by/4.0/).

hypothesis that hair cells of human origin may be derived by some of the supporting cells rather than a bi-fated progenitor cells. The study also suggests the presence of intermediate otic-lineage cells that give rise to the supporting cells; and also highlights specific regulation of ion channel activities and wnt signaling pathways in supporting cells and hair cells, respectively. Overall, the manuscript provides a first piece of clue to how human hair cells develop in an organoid model, which may have implication in human hair cell development and regeneration in vivo.

However, the bulk of the study is based on scRNA-seq analyses, some of the claims should be substantiated and clarified by additional analyses and immunostaining validations.

Response: Thank you very much for your unequivocal endorsement of our manuscript. Based on your suggestion, we have included immunofluorescence validation data.

Major comments:
1. Probably the most significant novelties of this study is to identify the intermediate oti-lineage that give rise to supporting cells, which then differentiate into hair cells. This is in contrast with the default idea that bi-fated progenitors can either differentiate into supporting cells or hair cells. However, if the supporting cells differeniate directly to hair cells, there should be a transitional population that express both well-established supporting cell markers and immature (or mature) hair cell markers. This may be true with careful analyses of the scRNA-seq data, but validation of these transitional population using immuno-fluorescent co-staining of these markers will be necessary.
Response: Using immunofluorescence, we found SPARCL1^{+} MYO6^{+} *SOX2*$^{ntdTomato+}$ and SPARCL1^{+} POU4F3^{+} *SOX2*$^{ntdTomato+}$ cells in vesicles at both d50 and d55 (Fig.4 D,E). This reveals the existence of cells expressing both supporting and hair cell markers in human inner ear organoids. However, our data do not exclude the possibility of hair cell differentiation not via supporting cells. We have included a description of the new data in the result (P13, L5-7) and discussion (P18, L6-11) sections.

2. The scRNA-seq was performed with FACS-ed Sox2+ cells, which is understandable but may miss out potential Sox2- sensory cells. The authors should discuss this in the text. In addition, the authors should specify in the Abstract that the hair cells are "vestibular type II-like hair cells", because it is unknown if vestibular type I or cochlear hair cells develop with the same trajectory.

Response: We previously reported that our human inner ear organoid protocol generates primarily type II vestibular hair cells (Koehler et al., 2017). We revisited this issue, as we had modified our protocol since our publication in 2017. In the d60 scRNA-seq dataset, we found expression of the vestibular hair cell markers *CD164L2*, *ZBBX*, *TEKT1*, *SKOR1*, *TCTEX1D1* and *NEUROD6*, but did not detect any of the cochlear markers *GATA3*, *INSM1*, *FGF8*, *LMOD3*, *LGR5*, *STRIP2*, and *SLC26A5* (Moore et al., 2022) in the hair cell cluster (Fig. S8). This strongly suggests that hair cells in this study are exclusively vestibular hair cells. Additionally, we confirmed expression of several type II hair cell markers, including *ANXA4*, *MAPT*, and *NHLH1* in the hair cell cluster; *CALB2* expression was low, but CALB2 was weakly positive in some hair cells by immunofluorescence. As for type I hair cell markers, we were unable to detect clear *SPP1* expression in the hair cell cluster, and unable to detect *SOX2*$^{ntdTomato-}$ type I hair cells (Stone et al., 2021, Lu et al., 2019) by immunofluorescence. Based on these results, we conclude that organoid hair cells in this study are mainly immature type II hair cells or immature vestibular hair cells. We have included these findings in the result section (P12, L7-12).

3. The finding that some of the hair cell markers (Myo6, Pcp4 etc) were expressed earlier than pioneer transcription factors such as Atoh1 and Pou4f3 is interesting, surprising and maybe thought provoking. Again, this should be experimentally validated by immuno-staining analyses of the p60 organoids to check if some of the hair cells express Myo6 etc but not Atoh1 or Pou4f3.

Response: Since there is no reliable ATOH1 antibody available, we used POU4F3 as a marker for pioneer transcription factors to compare the timing of early hair cell genes found in our

© 2023. Published by The Company of Biologists under the terms of the Creative Commons Attribution License (https://creativecommons.org/licenses/by/4.0/).

scRNA-seq data with that of POU4F3 expression (Fig. 4). We found that upregulation of MYO6 and CALM2 in hair cells precedes to that of POU4F3 (Fig. 4G,H). We have included these results (P13, L16 – P14, L2).

4. The presence of putative hair cells in d50 organoids is also interesting, how are they different from d60 in term of transcriptome? Are these d50 hair cells simply fate determined early or represent a distinct subtype of hair cells? A comparative analysis of d50 and d60 hair cells will be highly informative.

Response: Since the hair cell number in the d50 dataset (n = 35) was significantly smaller than that in the d60 dataset (n = 1,160), we were unable to perform trustable DEG analysis. Answering this question awaits future invstigation with a larger number of d50 samples/hair cells.

Minor comments:
1. Expression of Sox2 should also be included in the feature plots, to show if various cell types show differences in Sox2 expression.

Response: Unfortunately due to the insertion of the 2A-tdTomato-NLS-bGH polyA cassette as long as 1,767 bases after the *SOX2* coding region (Fig. 1A), we were unable to properly map *SOX2* sequencing data from 3' RNA- seq.

2. Fig. S4 and S5, NGFR and GAP43 are neuronal markers, which may show some expression in other cell types such as Schwann cells, however, they should be labelled as "neural markers" rather than "Schwann cell markers" to avoid confusion.

Response: We have corrected the annotation to "neural markers" in new Fig. S3 and Fig. S7 to avoid confusion based on the reviewer's suggestion.

3. Page 8, line 2, "maintained until d40", however, Fig. 1J shows d35.

Response: We have replaced the image in Fig. 1 for consistency with the statement.

<u>Reviewer 3</u>
Advance Summary and Potential Significance to Field:
Ueda et al. provide much-needed insight into the cellular lineages emerging in human inner ear organoid cultures. Inner ear (or otic) organoid cultures have high complexity relative to other organoid systems, so the application of single-cell approaches is needed to illuminate the diverse cell populations. Moreover, our knowledge of cell lineage decisions among otic epithelial cells—the key targets for inner ear therapies—is lacking. The authors aim to use these data to provide actionable insights for regenerative inner ear therapeutics. Here, the author's narrowed their analysis to SOX2+ cell populations from various time points between days 30-80, which limited the dataset to the sensory portions of the otic lineage (supporting cells and hair cells), neural crest cells, and some CNS-like cells. Overall, the analysis is thorough and well presented. The resulting data clarify the emergence of otic lineage cells in these cultures and largely reconfirm previous findings regarding the vestibular identity of organoids grown in this platform. A key weakness would be the lack of truly novel insights into the otic organoid culture cellular composition or the mechanisms of mammalian inner development, in general. Nonetheless, the dataset represents a valuable resource for anyone wishing to use otic organoids for their research.

Response: Thank you for your positive review of our manuscript and constructive and insightful comments.

Reviewer 3 Comments for
the Author: General:
As stated above, the analyses are thorough and executed following current bioinformatic standards. The visualizations are particularly well organized for readability.

© 2023. Published by The Company of Biologists under the terms of the Creative Commons Attribution License (https://creativecommons.org/licenses/by/4.0/).

Response: Thank you for your positive endorsement of our analyses and data presentation.

This study aimed to identify previously unrecognized genes or gene sets that are upregulated in early-stage human hair cell differentiation. The expression of ion channel and ion transporter-related proteins as well as the involvement of the WNT pathway is not surprising as this has been described during development. In revising the manuscript, the authors should highlight potentially novel insights. Do the data implicate other signaling pathways that have received less attention than the WNT pathway? An overview analysis of signaling pathway mediators expressed in the otic epithelial cells would be a good community resource. Moreover, the expressed goal of the study seemed to be to identify new transcription factors that may control otic development, yet these insights are lacking. A curated list of differentially expressed TFs could also be an excellent resource for the community. **Response:** We have listed all DEGs for the individual and merged datasets identified with the Seurat pipeline and highlighted TFs (Lambert et al., 2018) with bold fonts and asterisks (Fig. S6). We have highlighted only pathways relevant to hair cell development in Fig. 5 to avoid the figure becoming too busy. We have created a new supplementary table (Table S3) to list other pathways.

The authors use recent data on mouse inner ear development for comparison to human inner ear organoids, yet there is no discussion of the possible developmental differences between the mouse and human inner ear (on a scRNAseq level). In line with this, there is no validation of the RNAseq data: as in the confirmation of RNA/protein expression by IHC or ISH. Although a complete validation is unnecessary, some validation, particularly of novel markers would strengthen the manuscript.

Response: Based on the reviewer's suggestion, we performed immunofluorescence to confirm expression of otic lineage (PAX2 and FBXO2), neural (HUC/D (*ELAVL3/4*)), and hair cell (ANXA4, PCP4, and POU4F3) markers in d20, d40, and d60 (Fig. 1K,L and Fig. 2C-F) samples. Additionally, we stained for non-otic lineage (DACH1, LRP2, S100B, and S100A6; Fig. S5), and vestibular and cochlear markers (Moore et al., 2022) (ZBBX, CALB2, GATA3, INSM1; Fig. S8). Moreover, we identified cells exhibiting a transitional state between supporting cells and hair cells (MYO6^{+} SPARCL1^{+} and POU4F3^{+} SPARCL1^{+}; Fig. 4D,E) as well as those expressing the early hair cell markers MYO6 and CALM2 (Fig. 4K,L).

Concerns about accessibility: It's become typical with these types of studies to provide easy access to the data for readers less skilled in data science, either through a custom (CellxGene) or central (gEAR) web portal. The authors should use these resources to improve the impact of the study.

Moreover, spreadsheets containing the complete differential expression analysis, marker gene, and gene ontology data should accompany the paper.

Response: We have uploaded the merged dataset at the gEAR portal, which will become publically accessible at the time of manuscript acceptance. Additionally, we have included a list of DEGs for each population (Fig. S6) and the entire list of gene sets (Table S3).

Finally, the manuscript would benefit from a more targeted discussion of the weaknesses of the experimental design to aid the reader in interpreting the results in comparison to similar studies. For instance, the lack of biological replicates or alternate cell lines should be discussed. Was the depth of sequencing sufficient to address the author's questions? These factors do not devalue the work, but rather need to be stated clearly.

Response: We used *SOX2*$^{tdTomato+}$ cells collected from one culture batch for each dataset and only from the WA25 hESC line, although there were biological replicates (30-100+ aggregates per barch) and we excluded samples with low viability prior to sequencing (e.g. canceled sequencing several times). Moreover, the oldest dataset was d60, and we collected strong *SOX2*$^{tdTomato+}$ cells from d60 samples to increase a hair cell number for a successful trajectory analysis (Materials and Methods). These factors could have potentially skewed bioinformatics outputs, especially for the analyses of

© 2023. Published by The Company of Biologists under the terms of the Creative Commons Attribution License (https://creativecommons.org/licenses/by/4.0/).

non-sensory and non-otic cell types as well as more mature sensory cell types. It is also worth noting that Monocle is well established, but not perfect. The sequencing depth was set in accordance with the current recommendation for v3 of 3' sequencing (https://kb.10xgenomics.com/hc/en-us/articles/115002022743-What-is-the- recommended-sequencing-depth-for-Single-Cell-3-and-5-Gene-Expression-libraries-), and we believe that the resultant sequencing depths were sufficient for the trajectory analysis from otic progenitors to hair cells in this study; however, deeper scRNA-seq could potentially give a better resolution for otic development. We have included these insights in the discussion section (P22, L9 – P23, L2). We did try our best to obtain highest-possible quality data, and as both Flow core and Genomics core are located in the same building with our lab, we were able to minimize sample preparation time.

Minor:

6.12 The presence of type II vestibular hair cells is not shown in this manuscript, and there seems to be no effort to evaluate the types of hair cells using the RNA-seq data or histology. The authors should discuss this omission or include a deeper analysis of hair cell type.

Response: Although we previously showed that our differentiation protocol generates mainly type II vestibular hair cells (Koehler et al., 2017), we tested whether this is still recapitulated in this study. We found expression of the vestibular hair cell markers *CD164L2*, *ZBBX*, *TEKT1*, *SKOR1*, *TCTEX1D1*, and *NEUROD6*, but did not find any of the cochlear markers *GATA3*, *INSM1*, *FGF8*, *LMOD3*, *LGR5*, *STRIP2*, and *SLC26A5* (Moore et al., 2022) in the hair cell cluster, suggesting that the hair cells in our organoids are exclusively vestibular hair cells. Additionally, we confirmed expression of several type II hair cell markers, including *ANXA4, MAPT*, and *NHLH1* in the hair cell cluster; *CALB2* expression was low, but CALB2 was weakly positive in some hair cells by immunofluorescence. As for type I hair cell markers, we were unable to detect clear *SPP1* expression in the hair cell cluster, and unable to detect *SOX2*$^{ntdTomato-}$ type I hair cells (Stone et al., 2021, Lu et al., 2019) by immunofluorescence. Based on these results, we conclude that organoid hair cells in this study are mainly immature type II hair cells or immature vestibular hair cells that could give rise to type I or II hair cells. We have included findings in the result section (P12, L7-12).

7.2 The name of the hESC cell line used is not provided clearly.

Response: We have included the name of the parental hESC line we used to generate the *SOX2*-2A-ntdTomato line (WA25) (P7, L5). The name of the parental line is also listed in the Materials and Methods section.

Fig.1 L-L'': The SOX2 expression in the putative supporting cells is overexposed.

Response: We have reduced the intensity of the channel for *SOX2*^{ntdTomato}. For better presentation of hair cells, supporting cells, and neural projection, we have also changed the color assignment for each channel.

Fig.2 A: Please include the number of cells captured per timepoint.

Response: We have included the number of cells in individual datasets in the figure legend.

Fig.2 C: The putative endolymphatic duct population and immature dark cell population show very similar gene expression at d40. Are these cell types distinct? Furthermore, at d50-60 these populations are not shown. Please discuss.

Response: Since we focused on *SOX2*^{ntdTomato} expressing sensory cell populations, we were not able to adequately characterize other *SOX2*-negative cell populations surrounding sensory epithelia (Steevens et al., 2019, Gu et al., 2016). For instance, we found cells expressing the dark cell marker LRP2 in vesicles at d50, but they were *SOX2*$^{ntdTomato-}$ (Fig. S5D,E). We hope to answer the question in the future by analyzing *SOX2*-negative cell populations in inner ear organoids.

© 2023. Published by The Company of Biologists under the terms of the Creative Commons Attribution License (https://creativecommons.org/licenses/by/4.0/).

Fig.3 E: See above.

Response: We understand this suggestion for encouraging us to list the DEGs for each population. We hope the list of DEGs with highlighted TFs (Fig. S6) will satisfy your suggestion.

15.6 Vestibular identity of HCs is not shown in this manuscript.

Response: As we responded above, we believe that organoid hair cells in this study are exclusively vestibular hair cells.

17.3-5 Reword sentence for clarity.

Response: We have reworded the phrase according to the reviewer's suggestion.

19.5 Transitional epithelial cells and dark cells are distinct cell types. Merging of datasets: has an algorithm for the integration of datasets been used? For instance, Harmony or CCA? Were multiple methods tested to assess over- correction?

Response: No algorithm was used for the integration of the datasets. Instead, the merge function in Seurat was used to analyze the datasets for each time point. This method was employed because when CCA or other integration is employed, differences between similar cell types in adjacent time points were eliminated, making it impossible to plot a developmental trajectory and analyze changes over time. Although we do think that transitional cells and dark cells are different cell types, distinguishing those cell types in this study is incomplete because mature dark cells were excluded from our datasets due to the lack of SOX2 expression (Steevens et al., 2019, Gu et al., 2016); we found cells expressing the dark cell marker $LRP2^{+}$ in vesicles at d50, but these cells were $SOX2^{ntdTomato-}$ (Fig. S5). To avoid confusion in the manuscript, we rephrased the term "transitional epithelium" to "non-sensory epithelium" to explain our interpretation more precisely.

26.16 Missing symbols

Response: We understood the part was garbled when converting a Word file to a PDF. We have corrected this issue in the revised manuscript.

References

BENKAFADAR, N., JANESICK, A., SCHEIBINGER, M., LING, A. H., JAN, T. A. & HELLER, S. 2021. Transcriptomic characterization of dying hair cells in the avian cochlea. *Cell Rep,* 34, 108902.

BRAMHALL, NAOMI F., SHI, F., ARNOLD, K., HOCHEDLINGER, K. & EDGE, ALBERT S. B. 2014. *Lgr5*-Positive Supporting Cells Generate New Hair Cells in the Postnatal Cochlea. *Stem Cell Reports,* 2, 311-322.

GU, R., BROWN, R. M., HSU, C.-W., CAI, T., CROWDER, A. L., PIAZZA, V. G., VADAKKAN, T. J., DICKINSON, M. E. & GROVES, A. K. 2016. Lineage tracing of Sox2-expressing progenitor cells in the mouse inner ear reveals a broad contribution to non-sensory tissues and insights into the origin of the organ of Corti. *Developmental Biology,* 414, 72-84.

JAN, T. A., ELTAWIL, Y., LING, A. H., CHEN, L., ELLWANGER, D. C., HELLER, S. & CHENG, A. G. 2021. Spatiotemporal dynamics of inner ear sensory and non-sensory cells revealed by single-cell transcriptomics. *Cell Rep,* 36, 109358.

KOEHLER, K. R., NIE, J., LONGWORTH-MILLS, E., LIU, X. P., LEE, J., HOLT, J. R. & HASHINO, E. 2017. Generation of inner ear organoids containing functional hair cells from human pluripotent stem cells. *Nat Biotechnol,* 35, 583-589.

LAMBERT, S. A., JOLMA, A., CAMPITELLI, L. F., DAS, P. K., YIN, Y., ALBU, M., CHEN, X., TAIPALE, J., HUGHES, T. R. & WEIRAUCH, M. T. 2018. The Human Transcription Factors. *Cell,* 172, 650-665.

LU, J., HU, L., YE, B., HU, H., TAO, Y., SHU, Y., HAO, C., BORSE, V., XIANG, M., WU, H., EDGE, A. S. B. & SHI, F. 2019. Increased Type I and Decreased Type II Hair Cells after Deletion of Sox2 in the

© 2023. Published by The Company of Biologists under the terms of the Creative Commons Attribution License (https://creativecommons.org/licenses/by/4.0/).

Developing Mouse Utricle. *Neuroscience,* 422, 146-160.
MOORE, S. T., NAKAMURA, T., NIE, J., SOLIVAIS, A. J., ARISTIZABAL-RAMIREZ, I., UEDA, Y., MAYAKANNAN, M., VUMMIDI, S. R., ROMANO, D. R., PERRIN, B. J., NELSON, R., FROLENKOV, G., CHUVA DE SOUSA LOPES, S. M. & HASHINO, E. 2022. Generating High-Fidelity Cochlear Organoids from Human Pluripotent Stem Cells. *SSRN*.
ROCCIO, M. & EDGE, A. S. B. 2019. Inner ear organoids: new tools to understand neurosensory cell development, degeneration and regeneration. *Development,* 146.
SHU, Y., LI, W., HUANG, M., QUAN, Y.-Z., SCHEFFER, D., TIAN, C., TAO, Y., LIU, X., HOCHEDLINGER, K., INDZHYKULIAN, A. A., WANG, Z., LI, H. & CHEN, Z.-Y. 2019. Renewed proliferation in adult mouse cochlea and regeneration of hair cells. *Nature Communications,* 10, 5530.
STEEVENS, A. R., GLATZER, J. C., KELLOGG, C. C., LOW, W. C., SANTI, P. A. & KIERNAN, A. E. 2019. SOX2 is required for inner ear growth and cochlear nonsensory formation before sensory development. *Development,* 146.
STONE, J. S., PUJOL, R., NGUYEN, T. B. & COX, B. C. 2021. The transcription factor Sox2 is required to maintain the cell type-specific properties and innervation of type II vestibular hair cells in adult mice. *J Neurosci*.
WANG, T., CHAI, R., KIM, G. S., PHAM, N., JANSSON, L., NGUYEN, D. H., KUO, B., MAY, L. A., ZUO, J., CUNNINGHAM, L. L. & CHENG, A. G. 2015. Lgr5+ cells regenerate hair cells via proliferation and direct transdifferentiation in damaged neonatal mouse utricle. *Nat Commun,* 6, 6613.

Second decision letter

MS ID#: DEVELOP/2022/201071

MS TITLE: Defining developmental trajectories of prosensory cells in human inner ear organoids at single-cell resolution

AUTHORS: Yoshitomo Ueda, Takashi Nakamura, Jing Nie, Alexander J Solivais, John R Hoffman, Becca J Daye, and Eri Hashino

I have now received all the referees reports on the above manuscript, and have reached a decision. The referees' comments are appended below, or you can access them online: please go to BenchPress and click on the 'Manuscripts with Decisions' queue in the Author Area.

The overall evaluation is positive and we would like to publish a revised manuscript in Development, provided that the referees' comments can be satisfactorily addressed. As you can see, the comments are minor and meant to help clarify certain areas in the manuscript. Please attend to all of the reviewers' comments in your revised manuscript and detail them in your point-by-point response. If you do not agree with any of their criticisms or suggestions explain clearly why this is so. If it would be helpful, you are welcome to contact us to discuss your revision in greater detail. Please send us a point-by-point response indicating your plans for addressing the referee's comments, and we will look over this and provide further guidance.

Reviewer 1

Advance summary and potential significance to field

This is a strong manuscript in which the authors have established a human organoid system, and used it to examine the trajectory of the sensory lineage using SOX2-positive cells. Their data supports their novel hypothesis that hair cells may derive from supporting cells instead of a bipotential progenitor cell and they further demonstrate that Wnt-signaling is a critical factor in the generation of hair cells in their system.

© 2023. Published by The Company of Biologists under the terms of the Creative Commons Attribution License (https://creativecommons.org/licenses/by/4.0/).

Comments for the author

I am satisfied with the responses from the authors to the comments and critiques.

Reviewer 2

Advance summary and potential significance to field

The revised manuscript by Ueda and coauthors reports the trajectory analysis of human hair cell development in an organoid model. Combining scRNA-seq and immunofluorecence analyses at multiple stages, the authors found that the vestibular hair cells may develop from populations of supporting cells, in which ion channel and wnt activities play an important role. This study, along with previous papers from this lab, significantly advances our understandings of human hair cell development and demonstrates the uniqueness of organoid models in auditory research.

Comments for the author

Not all readers may be fully aware of the nature of the hair cells in this organoid system, I would still urge the authors to spell out the identity of the hair cells in the abstract. Specifically, "Our pseudotime analysis suggests that hair cells..." should be "Our pseudotime analysis suggests that the vestibular type II-like hair cells...". This is particular important as not to over generalize the claim.

Reviewer 3

Advance summary and potential significance to field

Overall, the authors did a commendable job responding to reviewer suggestions, and the manuscript is much stronger for the effort. However, there are a few additional points the authors should consider before publication:

Comments for the author

- Fig 2C Non-sensory annotations: These annotations should be softened if the authors cannot adequately characterize the putative endolymphatic or dark cell populations through multiple marker gene expression or IHC. The annotations should change to something more general, such as “non-sensory cells.” The authors can speculate about what they might be.

- Page 8, line 8: Please justify labeling these “non-otic epithelial cells.”
Could they be mesenchymal cells?

- Page 16, line 17 – The decline of SOX2 expression as a feature of Type I hair cells: This is an interesting discussion point and explains why the analysis on SOX2+ cells did not capture vestibular Type I hair cells. Please make this point more straightforward. However, there may be nascent Type I’s in this data set. For discussion of this point, the authors should refer to notable works from the recent pre-print literature that provide extensive single-cell analysis of human fetal and organoid inner ear tissue and identify Type I-like hair cells in organoids:

1. Yu, K. S. et al. Development of the Mouse and Human Cochlea at Single-Cell Resolution. Biorxiv 739680 (2019) doi:10.1101/739680.

2. Steinhart, M. R. et al. Defining Inner Ear Cell Type Specification at Single-Cell Resolution in a Model of Human Cranial Development. Ssrn Electron J (2021) doi:10.2139/ssrn.3974124.

3. Valk, W. H. van der et al. A Single-Cell Level Comparison of Human Inner Ear Organoids and the Human Cochlea and Vestibular Organs. Biorxiv 2022.09.28.509835 (2022) doi:10.1101/2022.09.28.509835.

© 2023. Published by The Company of Biologists under the terms of the Creative Commons Attribution License (https://creativecommons.org/licenses/by/4.0/).

- Fig 6 - WNT signaling data: The addition of the IWP2 treatment is welcome; however, the approach to quantification needs clarification. Was quantification performed on live-cell images of one side of the organoids? Or were the specimens cleared first before area measurement? If the latter, the fluorescent signal is likely highly variable across samples of this size, and irregularity—image depth would be poor. For instance, if you flipped over the IWP2-treated organoid in Fig 6 panel B, could it look like the CTRL specimen in panel A? These drawbacks seem inherent to the organoid model and must be clearly stated in the discussion or methods to aid the reader's interpretation. One mitigation strategy would be to include supplementary data showing 3-4 representative specimens per group to give the reader a sense of the irregularity across specimens.

Input on responses to other Reviewer's comments:

- Response to Reviewer 1 (2nd page, last paragraph): Using the name "intermediate cells" is potentially problematic. The authors should choose a name different from a pre-existing cell type in the inner ear (i.e., intermediate cells = stria vascularis; transitional cells = vestibular epithelial cells between dark cells and supporting cells).

- Response to Reviewer 2 (3rd page, last paragraph): As also suggested by Reviewer 1, it would indeed be interesting to define the cellular changes between D50 and D60. If it is impossible to compare HCs between these two timepoints perhaps "intermediate otic cells" at D50 could be compared with D60 HCs. For this Reviewer, these analyses are not essential but could provide additional insight lacking in the manuscript.

Second revision

Author response to reviewers' comments

Reviewer 1
Advance Summary and Potential Significance to Field: *This is a strong manuscript in which the authors have established a human organoid system, and used it to examine the trajectory of the sensory lineage using SOX2- positive cells. Their data supports their novel hypothesis that hair cells may derive from supporting cells instead of a bipotential progenitor cell, and they further demonstrate that Wnt-signaling is a critical factor in the generation of hair cells in their system.*

Reviewer 1
Comments for the Author:

I am satisfied with the responses from the authors to the comments and critiques.

Response: Thank you very much for your endorsement of our manuscript.

Reviewer 2
Advance Summary and Potential Significance to Field: *The revised manuscript by Ueda and coauthors reports the trajectory analysis of human hair cell development in an organoid model. Combining scRNA-seq and immunofluorecence analyses at multiple stages, the authors found that the vestibular hair cells may develop from populations of supporting cells, in which ion channel and wnt activities play an important role. This study, along with previous papers from this lab, significantly advances our understandings of human hair cell development and demonstrates the uniqueness of organoid models in auditory research.*

Response: Thank you very much for your positive feedback on our manuscript.

Reviewer 2 Comments for the Author:
Not all readers may be fully aware of the nature of the hair cells in this organoid system, I would still urge the authors to spell out the identity of the hair cells in the abstract. Specifically, "Our

© 2023. Published by The Company of Biologists under the terms of the Creative Commons Attribution License (https://creativecommons.org/licenses/by/4.0/).

pseudotime analysis suggests that hair cells..." should be "Our pseudotime analysis suggests that the vestibular type II-like hair cells...". This is particular important as not to over generalize the claim.

Response: We have revised and added the hair cell identities per the reviewer's suggestion.

Reviewer 3
Advance Summary and Potential Significance to Field: *Overall, the authors did a commendable job responding to reviewer suggestions, and the manuscript is much stronger for the effort. However, there are a few additional points the authors should consider before publication:*

Response: Thank you for your additional feedback for further discussion.

Reviewer 3 Comments for the Author:
Fig 2C Non-sensory annotations: These annotations should be softened if the authors cannot adequately characterize the putative endolymphatic or dark cell populations through multiple marker gene expression or IHC. The annotations should change to something more general, such as "non-sensory cells." The authors can speculate about what they might be.

Response: Based on the reviewer's comment, we have renamed those populations as "Otic nonsensory 1" and "Otic nonsensory 2". We have included these new annotations in Fig. 2, Fig. 3, Fig. 4, Fig. S3, and Fig. S6, and revised the manuscript accordingly.

Page 8, line 8: Please justify labeling these "non-otic epithelial cells." Could they be mesenchymal cells? **Response:** We erroneously labeled "non-otic epithelial cells". We have revised to the correct terminology, "non- otic cells".

Page 16, line 17 – The decline of SOX2 expression as a feature of Type I hair cells: This is an interesting discussion point and explains why the analysis on SOX2+ cells did not capture vestibular Type I hair cells. Please make this point more straightforward. However, there may be nascent Type I's in this data set. For discussion of this point, the authors should refer to notable works from the recent pre-print literature that provide extensive single-cell analysis of human fetal and organoid inner ear tissue and identify Type I-like hair cells in organoids:

1.Yu, K. S. et al. Development of the Mouse and Human Cochlea at Single-Cell Resolution. Biorxiv 739680 (2019) doi:10.1101/739680.

2.Steinhart, M. R. et al. Defining Inner Ear Cell Type Specification at Single-Cell Resolution in a Model of Human Cranial Development. Ssrn Electron J (2021) doi:10.2139/ssrn.3974124.

3.Valk, W. H. van der et al. A Single-Cell Level Comparison of Human Inner Ear Organoids and the Human Cochlea and Vestibular Organs. Biorxiv 2022.09.28.509835 (2022) doi:10.1101/2022.09.28.509835.

Response: We have included a discussion on challenges associated with type I hair cell identification with the current protocol (P22, L2-5), citing our previous report displaying evidence of type I hair cells in the current inner ear organoid system (Liu et al., 2016).

However, we have concerns about the suggested preprints as described below:

1. The scarce number of hair cells in the entire cell populations in *Steinhart, et al.* and *Valk, W. H. van der, et al.* impedes the significance of their conclusions drawn on hair cell development. The hair cell numbers in *Steinhart, et al.* are 10 (d18), 8, (d24), 14 (d30), and 34 (d36) in about 17,000 cells per each inner ear organoid dataset; the ones estimated in *Valk, W. H. van der, et al.* are about 10 in 22,742 cells and about 50 in 27,465 nuclei in scRNA-seq and snRNA-seq of inner ear organoids, respectively.

2. Extremely rare evident type I hair cells in *Steinhart, et al.* and *Valk, W. H. van der, et al.* hamper effective discussion on type I hair cell existence in inner ear organoids; *Steinhart, et al.*

© 2023. Published by The Company of Biologists under the terms of the Creative Commons Attribution License (https://creativecommons.org/licenses/by/4.0/).

contains only two *OCM*$^+$ putative type I hair cells in about 68,000 cells, and *Valk, W. H. van der, et al.* only shows a couple of faintly OCM$^+$ hair cells without any evidence in scRNA-seq or snRNA-seq data. Moreover, OCM is not a specific type I hair cell marker as previously described (McInturff et al., 2018).

3. *Yu, K. S., et al.* encompasses only cochlear cell populations with no vestibular cell types. We have cited *Valk et al.* in the introduction section (P6, L7), as this manuscript is most relevant to the present study.

Fig 6 - WNT signaling data: The addition of the IWP2 treatment is welcome; however, the approach to quantification needs clarification. Was quantification performed on live-cell images of one side of the organoids? Or were the specimens cleared first before area measurement? If the latter, the fluorescent signal is likely highly variable across samples of this size, and irregularity—image depth would be poor. For instance, if you flipped over the IWP2-treated organoid in Fig 6 panel B, could it look like the CTRL specimen in panel A? These drawbacks seem inherent to the organoid model and must be clearly stated in the discussion or methods to aid the reader's interpretation. One mitigation strategy would be to include supplementary data showing 3-4 representative specimens per group to give the reader a sense of the irregularity across specimens.

Response: We performed quantification using live-cell images, always selecting a side that displayed the broadest and strongest expression of *POU4F3*^{ntdTomato} in each aggregate. We have described these details in the materials and methods section. Based on the reviewer's suggestion, we have included several representative images in Fig. 6.

Input on responses to other Reviewer's comments:
Response to Reviewer 1 (2nd page, last paragraph): Using the name "intermediate cells" is potentially problematic. The authors should choose a name different from a pre-existing cell type in the inner ear (i.e., intermediate cells = stria vascularis; transitional cells = vestibular epithelial cells between dark cells and supporting cells).

Response: We have replaced the term "intermediate cells" with "intermediate otic cells" throughout the manuscript. We feel that "intermediate otic cells" is seldom confused with "intermediate cells" in the stria vascularis because our data do not include any cochlear cell types, such as stria vascularis cells.

Response to Reviewer 2 (3rd page, last paragraph): As also suggested by Reviewer 1, it would indeed be interesting to define the cellular changes between D50 and D60. If it is impossible to compare HCs between these two timepoints, perhaps "intermediate otic cells" at D50 could be compared with D60 HCs. For this Reviewer, these analyses are not essential but could provide additional insight lacking in the manuscript.

Response:
Comparing between the cell populations the reviewer suggested is, in our view, redundant to the analysis we performed for Figure S6 and does not add any new information to the manuscript. Technically we can generate a volcano plot for D50 intermediate otic cells vs. D60 hair cells, but the majority of the resultant differentially expressed genes between these cell populations are also found in the lists of differentially expressed genes for D50 intermediate otic cells and D60 hair cells (Fig. S6). Because of this reason, we have opted not to include the comparison. Thank you for your suggestion.

References
LIU, X. P., KOEHLER, K. R., MIKOSZ, A. M., HASHINO, E. & HOLT, J. R. 2016. Functional development of mechanosensitive hair cells in stem cell-derived organoids parallels native vestibular hair cells. *Nat Commun*, 7, 11508.
MCINTURFF, S., BURNS, J. C. & KELLEY, M. W. 2018. Characterization of spatial and temporal development of Type I and Type II hair cells in the mouse utricle using new cell-type-specific markers. *Biol Open*, 7.

© 2023. Published by The Company of Biologists under the terms of the Creative Commons Attribution License (https://creativecommons.org/licenses/by/4.0/).

Third decision letter

MS ID#: DEVELOP/2022/201071

MS TITLE: Defining developmental trajectories of prosensory cells in human inner ear organoids at single-cell resolution

AUTHORS: Yoshitomo Ueda, Takashi Nakamura, Jing Nie, Alexander J Solivais, John R Hoffman, Becca J Daye, and Eri Hashino
ARTICLE TYPE: Research Article

I am happy to tell you that your manuscript has been accepted for publication in Development, pending our standard ethics checks.

© 2023. Published by The Company of Biologists under the terms of the Creative Commons Attribution License (https://creativecommons.org/licenses/by/4.0/).
